# Ethnobotanical Survey on Bitter Tea in Taiwan

**DOI:** 10.3389/fphar.2022.816029

**Published:** 2022-02-18

**Authors:** Jung Chao, Ting-Yang Chen, Li-Heng Pao, Jeng-Shyan Deng, Yung-Chi Cheng, Shan-Yu Su, Shyh-Shyun Huang

**Affiliations:** ^1^ Chinese Medicine Research Center, Department of Chinese Pharmaceutical Sciences and Chinese Medicine Resources, Master Program for Food and Drug Safety, China Medical University, Taichung, Taiwan; ^2^ Chinese Medicine Research Center, Department of Chinese Pharmaceutical Sciences and Chinese Medicine Resources, China Medical University, Taichung, Taiwan; ^3^ Graduate Institute of Health Industry Technology, Research Center for Food and Cosmetic Safety, and Research Center for Chinese Herbal Medicine, College of Human Ecology, Chang Gung University of Science and Technology, Taoyuan, Taiwan; ^4^ Department of Gastroenterology and Hepatology, Chang Gung Memorial Hospital, Taoyuan, Taiwan; ^5^ Department of Food Nutrition and Health Biotechnology, Asia University, Taichung, Taiwan; ^6^ Department of Pharmacology, Yale University School of Medicine, New Haven, CT, United States; ^7^ Department of Chinese Medicine, China Medical University Hospital, School of Post-Baccalaureate Chinese Medicine, College of Chinese Medicine, China Medical University, Taichung, Taiwan; ^8^ School of Pharmacy, China Medical University, Taichung, Taiwan

**Keywords:** Taiwanese bitter tea, field survey, Taiwanese traditional medicine, ethnobotany, health geography

## Abstract

**Ethnopharmacological evidence:** In Taiwan, herbal tea is considered a traditional medicine and has been consumed for hundreds of years. In contrast to regular tea, herbal teas are prepared using plants other than the regular tea plant, Camellia sinensis (L.) Kuntze. Bitter tea (kǔ-chá), a series of herbal teas prepared in response to common diseases in Taiwan, is often made from local Taiwanese plants. However, the raw materials and formulations have been kept secret and verbally passed down by store owners across generations without a fixed recipe, and the constituent plant materials have not been disclosed.

**Aim of the study:** The aim was to determine the herbal composition of bitter tea sold in Taiwan, which can facilitate further studies on pharmacological applications and conserve cultural resources.

**Materials and methods:** Interviews were conducted through a semi-structured questionnaire. The surveyed respondents were traditional sellers of traditional herbal tea. The relevant literature was collated for a systematic analysis of the composition, characteristics, and traditional and modern applications of the plant materials used in bitter tea. We also conducted an association analysis of the composition of Taiwanese bitter tea with green herb tea (qing-cao-cha tea), another commonly consumed herbal tea in Taiwan, as well as herbal teas in neighboring areas outside Taiwan.

**Results:** After visiting a total of 59 stores, we identified 32 bitter tea formulations and 73 plant materials. Asteraceae was the most commonly used family, and most stores used whole plants. According to a network analysis of nine plant materials used in high frequency as drug pairs, Tithonia diversifolia and Ajuga nipponensis were found to be the core plant materials used in Taiwanese bitter tea.

**Conclusion:** Plant materials used in Taiwanese bitter tea were distinct, with multiple therapeutic functions. Further research is required to clarify their efficacy and mechanisms.

## Introduction

Herbal tea is a drink composed of plants other than *Camellia sinensis* (L.) Kuntze of the Theaceae family—in contrast to regular tea—and is prepared by decocting or brewing with hot water. Herbal tea is commonly prepared with local plants that can be easily obtained (Fu et al., 2018). The custom of brewing tea with herbs is found all over the world, including Europe (Soukand and Kalle, 2013; Soukand et al., 2013), America (Joubert et al., 2008), Africa (Roulette et al., 2018), and Asia (Hu, 2005). The herbs used could be a single herb or a mixture of multiple plants. In many areas, herbal teas are used as therapeutic vehicles to treat associated health conditions (Poswal et al., 2019).

In China, herbal tea has been consumed for more than 2000 years (Liu et al., 2013). Famous herbal teas in pan-China include those are drunk in Yao area (Jin et al., 2018), Lingnan (Liu Y. et al., 2013), Chaoshan (Li et al., 2017), and Fujian (Lin, 2014), and Taiwan (Chang, 2005). Furthermore, this beverage has been closely associated with the prevention and treatment of local common ailments (Li et al., 2017; Tan et al., 2017). The herbal teas in Lingnan (Liu Y. et al., 2013), Chaoshan (Li et al., 2017), and Fujian (Lin, 2014) are called the“cool tea,” implying that the herbal teas are used against the hot weather in southern China. The type of herbal tea is also influenced by the traditional culture of various regions, thereby reflecting local characteristics (Hu, 2005).

Although the folk plants in Taiwan are widely used in religious rites, bathing, cuisine, and herbal tea, international literature on Taiwanese folk plants is limited. According to previous surveys, there are 1,217 wild or cultivated folk plants with medicinal purposes have been documented in Taiwan (Ministry of Health and Welfare, 2021). The two main herbal teas in Taiwan, bitter tea (kŭ-chá) and green herb tea (qīng-căo-chá), are the two most complicated applications of folk plants in Taiwan. Both of them are usually made by cooking a mixture of medicinal plants (Chang, 2004). The mixtures of medicinal plants, in which there are the mixtures of bioactive compounds, exert synergistic therapeutic effects (Gertsch, 2011; Gras et al., 2018). Herbal teas in Taiwan are believed to have originated in southern China. After arriving in Taiwan, the ingredients of original herbal teas gradually turned into readily available Taiwanese native plants, and then, herbal teas that are suitable for local people were gradually developed (Chen and Lin, 2012).

Although the formulations of bitter tea and green herb tea vary across Taiwanese stores, they are all primarily advertised as having the capacity to “clear heat” (Huang et al., 2020). In the principle of traditional Chinese medicine formulation, one to three medicinal materials play the role of core medicinal materials in an herbal mixture. Other medicinal materials are added to enhance the therapeutic effects, improve the taste, or reduce the side effects of the core plant medicinal materials (Zhou et al., 2016).

According to previous research and surveys, the main components of green herb tea in Taiwan are *Platostoma palustre* (Blume) A.J.Paton, *Bidens pilosa* L., *Pteris multifida* Poir., *Mentha arvensis* L., *Sphagneticola calendulacea* (L.) Pruski, and *Rhinacanthus nasutus* (L.) Kurz (Huang et al., 2020). These tea beverages prepared by mixing and decocting these plants have a refreshing, half bitter/half sweet taste, and are used for quenching thirst and relieving summer heat (Huang et al., 2020). The other Taiwanese main herbal tea, the Taiwanese bitter tea, is also known as liver nourishing tea (yăng-gān-chá), and thick green herb tea (nóng-hóu-qīng-căo-chá). It is characterized by a bitter-dominated taste that is stronger than that of regular green herb tea (Chang, 2005). Although bitter tea has been consumed for hundreds of years in Taiwan, its components and core medicinal materials have not been comprehensively studied, which has impeded the investigation of its efficacy against various health conditions.

Traditional Chinese medicine categorizes medicinal materials according to their property and flavor. The property includes hot, warm, neutral, cool, cold. Hot and warm materials are usually taken to supply energy to the body, while cool and cold materials are taken to drain away the heat from the body (Liu et al., 2020). The flavors are divided into sour, bitter, sweet, spicy, salty, and plain. Flavor is the taste of herbs in the mouth. Each of the flavors has distinct medicinal therapeutic functions. Sour herbs astringe the leakage of fluids and energy; sweet herbs tonify and harmonize; spicy herbs disperse and move; salty herbs soften and purge; plain herbs leech out dampness; and finally, the traditional therapeutic function associated with bitter herbs is heat clearing and removing dampmess (Bensky et al., 2004; Lu et al., 2018; Liu et al.,. 2020). The heat-clearing and dampess-draining effects provided by the bitter taste are used to combat the discomfort caused by the summer rainy season (Wu, 2005). Moreover, according to a previous study, the administration of bitter drugs can help treat liver disease (Tsai et al., 2020). For example, *Gentiana scabra* Bunge, *Artemisia capillaris* Thunb., and *Scutellaria baicalensis* Georgi are the most classical and commonly used traditional Chinese medicines for liver disease (Tsai et al., 2020).

Only one of the two main types of herbal tea in Taiwan, the composition and pharmacology of green herb tea constituents have been studied and reported (Huang et al., 2020), whereas those of the raw materials of bitter tea have not yet been elucidated. According to local reference books, most raw materials used in such herbal teas are either cultivated locally or wildly harvested. They require simple process after collection and are decocted after drying (Chang, 2005). However, the formula of Taiwanese bitter tea is not in the public domain, considering that every store keeps their recipe a secret. In the present study, we surveyed sellers of folk herbal medicine in Taiwan. The survey was conducted via interviews using semi-structured questionnaires, and information recorded included the current status of the bitter tea market in Taiwan as well as the ingredients and formulations used. The results of the present study can help reveal the ethnopharmacological aspects of bitter tea consumed in Taiwan and facilitate the conservation of such unique cultural resources.

## Materials and Methods

### Survey Area and Period

**FIGURE 1:**
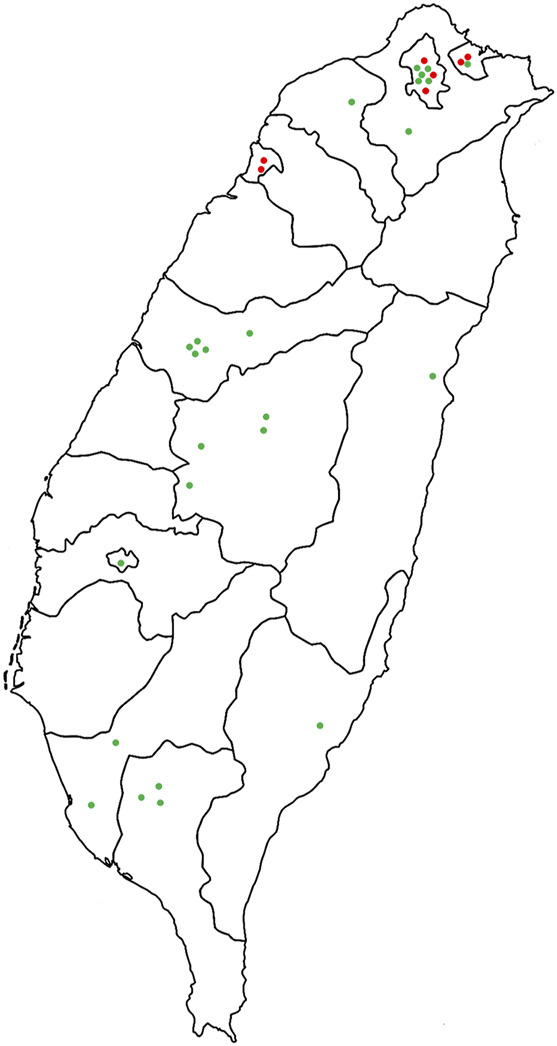
Geographical distribution of 32 stores with available bitter tea formulations. Red dots indicate that the store provided bitter tea formulation but did not receive interviews (seven stores), and green dots indicate that the store provided complete bitter tea formulation and accepted interviews (25 stores).

Taiwan, an island in East Asia, is located at 21°45′-25°56′N and 119°18′-124°34′E. It spans the Tropic of Cancer, covers an area of 36,197 km^2^, and mostly has a subtropical climate with monsoons, warm and humid summers, and heavy rainfall. In the present study, 59 stores were visited, 25 of which provided information on bitter tea formulations and participated in the interviews. Seven stores only provided information on the bitter tea formula used in their stores but did not give interviews. The 32 sampled stores were distributed all over Taiwan (Figure 1). The survey period was from December 2018 to April 2019, and the entire research activity was reviewed and approved by Taiwan’s Central Regional Research Ethics Center (CRREC-107-019) (Supplementary Figure S1).

### Interviews and Data Collection

A semi-structured questionnaire was used for interviews, and the subjects were store owners selling bitter tea. The stores were found online or introduced to us by organizations involved with local medicinal plants. The store owners were invited to participate in the survey, and interviews were conducted on a voluntary basis. With the permission of the informants, written records, audio recordings, and photographs were obtained, and herbal medicines were acquired by the authors. The medicinal materials were then identified using the five senses identification method, with which the expert identify the origin of medicinal materials by touch, taste, smell, hearing, and visual observation, without any equipments (Hsieh et al., 2017). The specimens of the medicinal materials have been deposited in the herbarium at School of Pharmacy of China Medical University (CMU) in Taichung City, Taiwan.

The information collected by the interviews was divided into two parts. The first part included basic information on the store, including age and sex of the store owner, store location, and how long the store has been in operation. The second part was a semi-structured questionnaire consisting of the following questions: 1) what is the composition of bitter tea; 2) what is the traditional therapeutic indication of the bitter tea sold in your store; 3) how was the traditional knowledge of the bitter tea obtained; and 4) does the store sell dry or fresh raw materials needed to make the tea?

### Data Collection of Bitter Tea Plant Materials in Taiwan

All data on the surveyed plant materials were collated, which included the following:

(1) Name, including scientific name, family name, and local name. The scientific and family names of plants are presented according to the Plant List nomenclature (The Royal Botanic Gardens, 2013) and the Plants of the World Online (Royal Botanic Garden, 2021).
(2) Part of the plant used. This was determined by identifying the raw plant materials.
(3) Frequency and use value (UV). Frequency refers to the number of stores using the plant material in the survey, and UV refers to the number of stores using the plant material/total number of stores in the survey as follows (Fu et al., 2018):

$$\text{UV}=\frac{\sum \text{Ui}}{N}$$

Where Ui represents the individual number of the *i*th medicinal material, and N represents the total number of bitter tea formulations.

(4) Traditional therapeutic functions. This information was obtained from the Committee on Chinese Medicine and Pharmacy (2003) and the second edition of The Committee on Chinese Medicine and Pharmacy (2011).
(5) The diversity of the medicinal plants was evaluated using Shannon diversity index (Gras et al., 2018), which was calculated as follows:

$$\text{Shannon diversity index}=-\overset{n}{\underset{i=1}{\sum }}Pi*log_{2}Pi$$

Where *Pi* represents the proportion of the individual number of the *i*th medicinal material to the individual number of the total medicinal material. For example, *Andrographis paniculata* was used in 10 of the formulations, and the total number of individual of all species in this survey is 183. Then the *Pi* for *Andrographis paniculata* was obtained by dividing 10 by 183. Then the $"Pi*log_{2}Pi"$ for all the medicinal materials in the survey were summed up to get the Shannon diversity index.

(6) Modern pharmacology. Information was obtained by searching for the plant materials in the literature using the PubMed database^1^. Those cited in studies published before April 2021 and at a frequency greater than nine were included.
(7) Comparison between Taiwanese bitter tea and Taiwanese green herb tea. Based on the findings of Huang et al. (2020), we analyzed the similarities and differences between the plant materials used for each tea and further compared differences in flavor, UV, and application in modern pharmacology.
(8) Comparison of herbal tea composition across three regions in southern China. By referring to relevant published articles (Liu et al., 2013; Lin, 2014; Li et al., 2017), a Venn diagram was plotted using an online tool^2^ to analyze the relationships between Taiwanese bitter tea and herbal tea from Lingnan, Chaoshan, and Fujian.

### Core Network Analysis of Bitter Tea Use in Taiwan

Core network analysis was carried out using the Traditional Chinese Medicine Inheritance Support System (TCMISS) v2.5 (Wu et al., 2019; Chao et al., 2020; Wu Z. et al., 2020). Several matching frequency conditions were input into the software to find a suitable network diagram. Establishing connections when combinations of plant materials had a matching frequency greater than four was found the most proper to generate the network that clearly present the core medicinal materials. The length of the connection indicates the degree of matching frequency, and the size of the circles represent the relative UV.

## Results

### Respondents’ Data and Store Information

A total of 59 stores selling bitter tea were visited for this study. Among them, 25 participated in interviews and provided the formulations for bitter tea; 7 were unwilling to participate in the interviews but provided bitter tea formulations; and 27 declined to participate in interviews and did not provide formulations. Of the 25 traditional stores that participated in interviews, 50% have been operating for more than 50 years. The store owners were mainly male (76%), with ages ranging from 31 to 70 years. Knowledge on bitter tea formulations was mostly passed down through family members (76%) or apprentices (20%). All stores sold prepared bitter tea drinks (100%), 36% sold dry raw materials, and 48% sold both dry and fresh raw materials (Supplementary Figure S2). In addition, all bitter tea sellers sold green herb tea.

### Plant Materials Used in Bitter Tea and Their Territories in Taiwan

**FIGURE 2:**
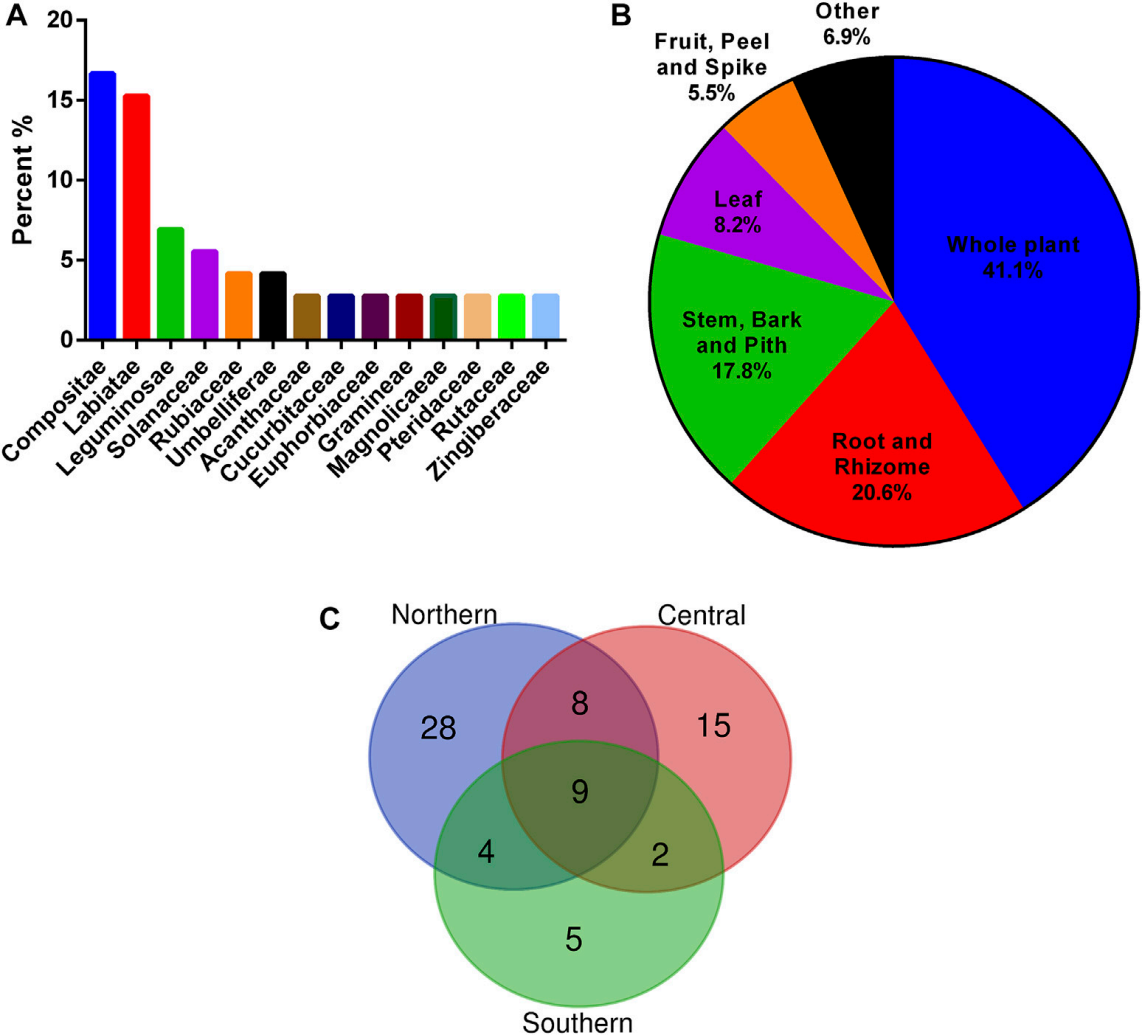
Origin analysis of 73 plant materials used in bitter tea. **(A)** Families; **(B)** plant parts; **(C)** the number of shared plant materials in areas of Taiwan.

In the present study, 73 plant materials from 72 plants belonging to 33 families and 67 genera were identified. Among the 73 medicinal materials, 60% of them were cultivated, 63% of them can be collected in the wild, and 37% were both cultivated and wild (Supplementary Table S1). The average number of plants materials in a formulation is 5.7 (Supplementary Table S2). The top five ranking species include *Andrographis paniculata* (Burm. f.) Nees, *Tithonia diversifolia* (Hemsl.) A. Gray (both UV = 0.312), *Ajuga nipponensis* Makino, *Ixeris chinensis* (Thunb. ex Thunb.) Nakai (both UV = 0.281), and *Ilex asprella* (Hook. & Arn.) Champ. ex Benth (UV = 0.25). Regarding the plant families, Asteraceae was the most frequently used, comprising 16.7% among 72 plants and 75.0% among 32 formulations, followed by Lamiaceae, comprising 15.3% among total plants and 65% among formulations (Figure 2A). The most used plant parts were the whole plant (41.1%), followed by the root and rhizome (20.6%) (Figure 2B).

The Shannon diversity index for all the 73 medicinal materials in Taiwan was 5.73. Among the northern, central, and southern Taiwan, the medicinal materials used in northern Taiwan were the most diverse, with a Shannon diversity index of 5.27. While the Shannon diversity indices of central and southern Taiwan were 4.92 and 4.11, respectively. Moreover, the more the north, the more species of medicinal materials were used. A total of 49 medicinal materials were used in bitter tea in northern Taiwan, while there were only 34 medicinal materials used in Central Taiwan, and even fewer in southern Taiwan, with only 20 medicinal materials (Supplementary Table S3). In these three areas, only nine medicinal materials were commonly used (Figure 2C). They were *Tithonia diversifolia* (stem)*, Ixeris chinensis, Rhinacanthus nasutus* (L.) Kurz*, Orthosiphon aristatus* (Blume) Miq.*, Andrographis paniculata* (Burm. f.) Nees*, Solanum incanum, Mallotus repandus* (Willd.) Muell.-Arg.*, Sphagneticola calendulacea* (L.) Pruski*,* and *Pteris multifida* Poir.

### Ethnomedicinal Functions of the Bitter Tea

When asked about the traditional applications of the bitter tea sold at a store, all sellers answered based on traditional concepts in Taiwan folk therapy; the bitter flavor is said to protect the liver, and the main ethnomedicinal function of bitter tea is “clearing heat, protecting the liver, and lowering liver fire.” The sellers also stated that bitter tea is a type of folk therapy product for the liver.

**FIGURE 3:**
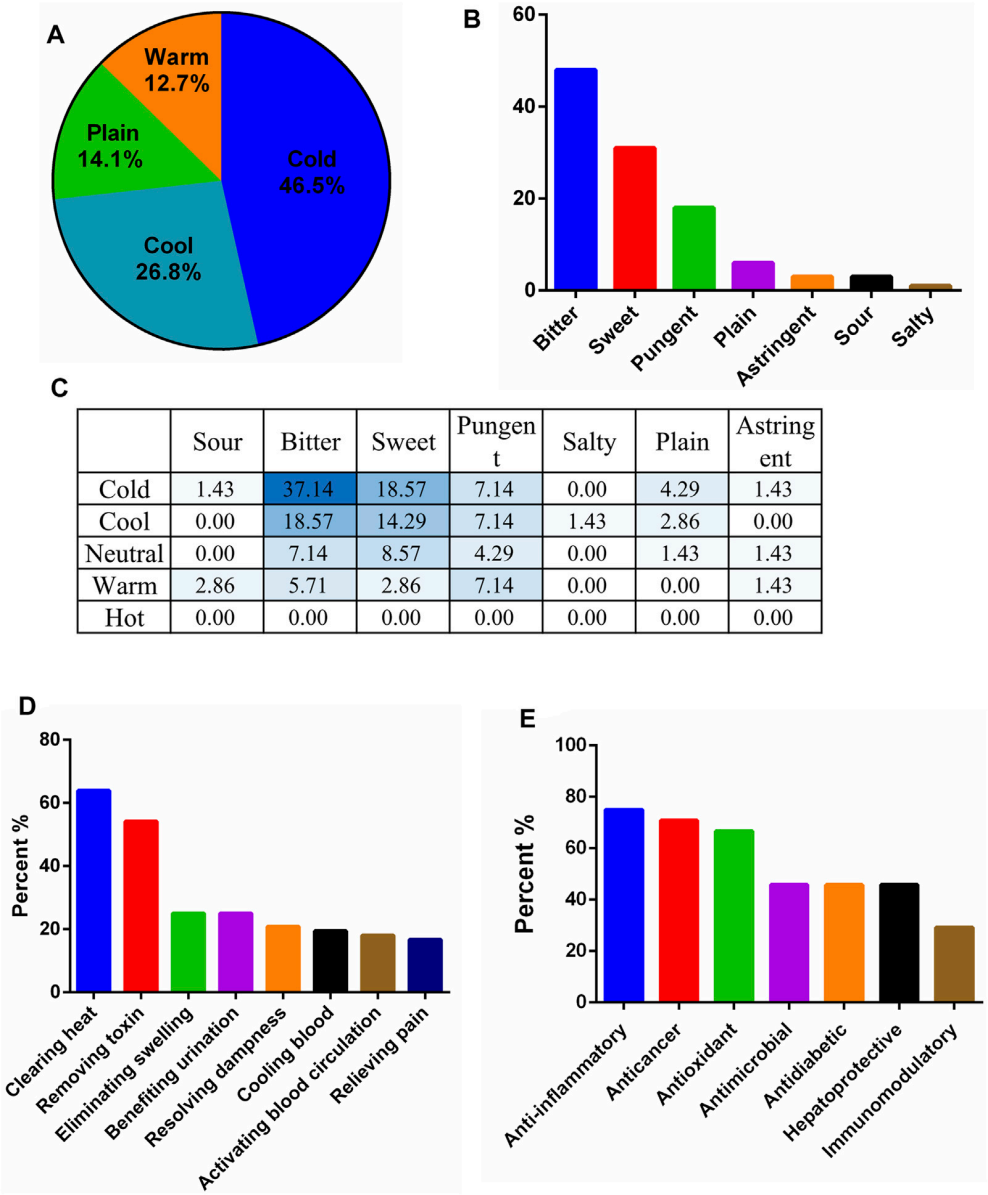
Traditional and modern pharmacological analysis of plant materials used in Taiwanese bitter tea. **(A)** Nature; **(B)** flavor; **(C)** comprehensive analysis of nature and flavor (%); **(D)** ethnomedicinal effects; **(E)** modern pharmacological applications of plant materials with a frequency greater than nine.

The traditional applications of the 73 identified plant materials were revealed, excluding *T. diversifolia* (stem) and *A. keiskei* since there were no records on their properties and flavors (Supplementary Table S1). The cold, hot, warm, and cool properties were then analyzed for 71 plant materials; cold properties were the most common (46.5%) followed by cool (26.8%), jointly accounting for 73.3% of the plant material properties (Figure 3A). Moreover, 69% of the plant materials had a bitter flavor (Figure 3B). According to a comprehensive analysis of the four properties and five flavors, cold and bitter plants are the most commonly used for preparing bitter tea in Taiwan (Figure 3C). Analysis of the ethnomedicinal function showed that 63.9, 54.8, 25.0, and 25.0% of the 73 plant materials had heat-clearing, detoxification, detumescence, and diuresis effects, respectively (Figure 3D).

Among the 24 commonly used plant materials in Taiwanese bitter tea, those with a frequency of use greater than nine were collected to analyze their modern pharmacological applications (Table 1). Anti-inflammation was the most common pharmacological action (Figure 3E), followed by anticancer, antioxidant, antimicrobial, hepatoprotection, and antidiabetic effects.

**TABLE 1:** Modern pharmacological effects and traditional use of highly cited plant materials used in Taiwanese bitter tea (UV > 0.09).

Voucher specimen numberCMUBT-X^a^	Scientific name	Family	Local name	Use value (%)	Related research on PubMed	Traditional use
1	*Andrographis paniculata* (Burm. f.) Nees	*Acanthaceae*	Chuān xīn lián (穿心蓮)	0.31	Antioxidant Mussard et al. (2020), anti-inflammatory Burgos et al. (2020), anti-hyperglycemic Wediasari et al. (2020), hepatoprotective Toppo et al. (2021), antimicrobial Hossain et al. (2021), anticancer Malik et al. (2021), immunomodulatory Liu et al. (2020), cardioprotective Lin et al. (2020), anti-hyperlipidemic, sexual functions and contraceptive Hossain et al. (2014), antiviral Jadhav and Karuppayil, (2021), Sa-Ngiamsuntorn et al. (2021), anti-snake venom Nayak et al. (2021), antimalarial Hassan et al. (2019), anti-Alzheimer Lu et al. (2019), and anti-obesity Chen et al. (2020)	Clears heat, resolves toxins, disperses swelling, and relieves pain
2	*Tithonia diversifolia (Hemsl.)* A. Gray (stem)	*Asteraceae (Compositae)*	Wǔ zhǎo jīn yīng (五爪金英)	0.31	Anti-inflammatory Broering et al. (2019), antimicrobial, antioxidant Ferreira Farias et al. (2019), anticancer Lu et al. (2017), analgesic Owoyele et al. (2004), anti-hyperlipidemic, antidiabetic Miura et al. (2005), antiviral Maregesi et al. (2010), gastroprotective Sanchez-Mendoza et al. (2011), immunomodulatory Ejelonu et al. (2017), antimalarial Afolayan et al. (2016), hepatoprotective Nguepi et al. (2021), anti-obesity, antiemetic, anti-diarrheal, anti-leishmania, and anti-trypanosoma Mabou Tagne et al. (2018)	-b
3	*Ajuga nipponensis* Makino	*Lamiaceae*	Bái mǎ wú gōng (白馬蜈蚣)	0.28	Antioxidant, hepatoprotective Hsieh et al. (2016), and anti-osteoclastogenic Wang, H. et al. (2021)	Disperses inflammation, cools the blood, and joins bone (Jiēgǔ)
4	*Ixeris chinensis* (Thunb.) Nakai	*Asteraceae (Compositae)*	Xiǎo jīn yīng (小金英)	0.28	Anticancer Xu et al. (2016), antiviral, hepatoprotective Shih et al. (2014), immunomodulatory Li et al. (2020), and antileukemic Chiang et al. (2004)	Clears heat, promotes urination, and calms the spirit
5	*Ilex asprella* (Hook. & Arn.) Champ. ex Benth	*Aquifoliaceae*	Wàn diǎn jīn (萬點金)	0.25	Anti-inflammatory Yang et al. (2018), anticancer Li et al. (2018), immunoregulatory Meng et al. (2018), lung-protective Dai et al. (2014), anti-hyperlipidemic Hu et al. (2012), and antiviral Zhang et al. (2018)	Clears heat, promotes urination, and calms the spirit
6	*Bombax ceiba* L.[*Bombax malabaricum* DC.]	*Malvaceae[Bombacaceae]*	Mù mián gēn (木棉根)	0.22	Antidiabetic Bhargava and Shah (2020), antioxidant Komati et al. (2020), anti-inflammatory, antisteatotic Arafa et al. (2019), promotes osteoblast proliferation Chauhan et al. (2018), antimicrobial Shah et al. (2018), hepatoprotective Lee et al. (2017), anticancer Tundis et al. (2014), anti-obesity Gupta et al. (2013), promotes male sexual function, and antiviral Wang et al. (2013)	Dispels wind, stops itchiness, dispels wind-dampness, clears heat, resolves toxins, removes stasis, and relives pain
7	*Solanum incanum* L	*Solanaceae*	Huáng shuǐ qié(黃水茄)	0.19	Anticancer Wu, Y.H. et al. (2015), Yu et al. (2017), Al-Emam et al. (2018) and antimicrobial Lashin et al. (2021)	Disperses inflammation, resolves toxins, dispels wind, relieves pain, clears heat, and disperses inflammation
8	*Mallotus repandus* (Willd.) Muell.-Arg	*Euphorbiaceae*	Tǒng jiāo téng (桶交藤)	0.16	Analgesic Hasan et al. (2014), anti-inflammatory Hasan et al. (2014), antioxidant Lin et al. (1995), and hepatoprotective Mondal et al. (2020)	Clears heat, resolves the exterior, benefits the throat, prevents rashes, improves digestion, disperses swelling, and stops itch
9	*Physalis angulata* L	*Solanaceae*	Dēng long cǎo (燈籠草)	0.16	Immunomodulatory Vieceli et al. (2021), anti-inflammatory Sun, C.P. et al. (2017a), Sun, C.P. et al. (2017b), Wang, L. et al. (2021), antiproliferative Sun, C.P. et al. (2017b), Chairissy et al. (2019), antioxidant Adewoye et al. (2016), reno-restorative Adewoye et al. (2016), antiparasitic Meira et al. (2015), antileishmanial Da Silva et al. (2018), and anticancer Ma et al. (2017)	Clears heat, resolves toxins, disperses swelling, and removes stasis
10	*Rhinacanthus nasutus* (L.) Kurz	*Acanthaceae*	Bái hè líng zhī(白鶴靈芝)	0.16	Anticancer Siripong et al. (2006), Kupradinun et al. (2009), Siripong et al. (2009), Horii et al. (2012), Siripong et al. (2012), Boueroy et al. (2018), anti-obesity Ngoc et al. (2019), anti-glycation Shah et al. (2017), neuroprotective Chuang et al. (2017), acetylcholinesterase inhibitor Brimson and Tencomnao, (2011), Brimson et al. (2012), Chang, C.Z. et al. (2016), Boonyaketgoson et al. (2018), antioxidant Brimson et al. (2012), Shah et al. (2017), Zhao et al. (2019), antidiabetic Shah et al. (2017), Visweswara Rao et al. (2013a), neuraminidase inhibitor Kwak et al. (2018), hepatoprotective Visweswara Rao et al. (2013b), anti-inflammatory Zhao et al. (2019), antimicrobial Kernan et al. (1997), Puttarak et al. (2010), Ngoc et al. (2019), anti-allergic Tewtrakul et al. (2009), immunomodulatory Punturee et al. (2005), anti-Parkinson’s Saleem et al. (2021), and antifungal Jeenkeawpieam et al. (2020)	Moistens the lung and stops coughing, calms the liver and reduces fire, disperses swelling, resolves toxins, kills worms, and stops itchiness
11	*Sigesbeckia orientalis* L	*Asteraceae (Compositae)*	Kǔ cǎo (苦草)Xī liàn cǎo (豨薟草)	0.16	Antimicrobial, anti-allergic, antithrombotic Wang, Q. et al. (2021), anti-inflammatory Nguyen et al. (2017), Chu et al. (2018), Engels et al. (2020), antihyperuricemic Nguyen et al. (2017), analgesic Nguyen et al. (2017), and anticancer Sun and Wang, (2006), Chang, C.C. et al. (2016)	Dispels wind-dampness and benefits sinew and bone
12	*Boehmeria nivea* (L.) Gaudich.[*Boehmeria nivea* (L.) Gaudich. var. *tenacissima* (Gaudich.) Miq.]	*Urticaceae*	Shān zhù má(山苧麻)	0.13	Anti-inflammatory Lim et al. (2020), laxative, antioxidant Lee et al. (2020), and antiproliferative Wang et al. (2019)	
13	*Glycyrrhiza uralensis* Fisch	*Leguminosae*	Gān cǎo (甘草)	0.13	Anticancer, antiulcer, spasmolytic, hepatoprotective, anti-inflammatory, antimicrobial Jiang et al. (2020), and anti-allergic Fouladi et al. (2019)	Supplements the spleen, boosts qi, relaxes tension, relieves pain, moistens lungs, stops coughing, drains fire, resolves toxins, and harmonizes the activity of other medicines
14	*Orthosiphon aristatus* (Blume) Miq	*Lamiaceae*	Huà shí cǎo (化石草)	0.13	Genoprotective Al-Dualimi et al. (2018), antibacterial Al-Dualimi et al. (2018), antidiabetic Damsud et al. (2014), antioxidant Hsu et al. (2010), anti-inflammatory Hsu et al. (2010), and antihypertensive Matsubara et al. (1999), Ohashi et al. (2000)	Clears heat, promotes urination, and clears kidney stones
15	*Platostoma palustre* (Blume) A.J.Paton [*Mesona chinensis* Benth.]	*Lamiaceae*	Xiān cǎo (仙草)	0.13	Antidiabetic Adisakwattana et al. (2014), Chusak et al. (2014), Liu S. et al. (2018), Yuris et al. (2019) and antioxidant Huang et al. (2019), Huang, L. et al. (2020)	Clears heat and resolves toxins
16	*Bidens pilosa* L.[*Bidens pilosa* L. var. *radiata* Sch. Bip.]	*Asteraceae (Compositae)*	Xián fēng cǎo (咸豐草)	0.09	Anticancer Arantes et al. (2021), gastroprotective Alvarez et al. (1999), Horiuchi et al. (2010), Anti-diabetic Chien et al. (2009), Ubillas et al. (2000) Anti-allergy Matsumoto et al. (2009), anti-inflammatory Hong et al. (2021), Xin et al. (2021) Anti-malaria Nadia et al. (2020) Anti-microbial Chiavari-Frederico et al. (2020) anti-coccidial, Yang et al. (2019), hepatoprotective Pegoraro et al. (2021), and anti-hypertensive Bilanda et al. (2017)	Clears heat, resolves toxins, promotes urination, and reduces jaundice
17	*Elephantopus scaber* L	*Asteraceae (Compositae)*	Ding shù wū(丁豎杇)	0.09	Anticancer Bai et al. (2020), Pandey et al. (2020), hepatoprotective Sulistyani and Nurkhasanah, (2020), anti-inflammatory Fu et al. (2020), Qi et al. (2020), antioxidant Aslam et al. (2016), antidiabetic, antimicrobial, and analgesic Hiradeve and Rangari, (2014)	Clears heat, resolves toxins, promotes urination, and disperses swelling
18	*Momordica charantia* L.[*Momordica charantia* L. var. *abbreviata* Ser.]	*Cucurbitaceae*	Shān kǔ guā(山苦瓜)	0.09	Anti-obesity Fan et al. (2021), anti-fatigue Hsiao et al. (2017), anti-inflammatory Perera et al. (2021), Tsai et al. (2016), antioxidant Akyuz et al. (2020), cell-protective Tsai et al. (2014), antimelanogenic Tsai et al. (2014), antidiabetic Kulkarni et al. (2021), and anticancer Ehigie et al. (2021)	Dispels wind, clears heat, clears the liver, and brightens the eyes
19	*Mucuna macrocarpa* Wall	*Leguminosae*	Xiě téng (血藤)	0.09	Antileukemic Lu et al. (2010)	Cures rheumatic pain (backache)
20	*Oldenlandia diffusa* (Willd.) Roxb.[*Hedyotis diffusa* Willd.]	*Rubiaceae*	Bái huā shé shé cǎo (白花蛇舌草)	0.09	Anticancer Chung et al. (2017), immunomodulatory, antioxidant Chen et al. (2016), and anti-inflammatory Zhu et al. (2018)	Clears heat, resolves toxins, engenders fluid, relieves thirst, and invigorates the blood
21	*Pteris multifida* Poir	*Pteridaceae*	Fèng wěi cǎo (鳳尾草)	0.09	Anti-cancer Kim et al. (2017), anti-neuroinflammatory Kim et al. (2016), anti-hyperlipidemic Wang et al. (2010), free radical-scavenging activity Wang et al. (2007), and anti-inflammatory Yin et al. (2018)	Clears heat, promotes urination, cools the blood, and resolves toxins
22	*Scutellaria barbata* D. Don	*Lamiaceae*	Bàn zhī lián (半枝蓮)	0.09	Anti-inflammatoryAkyuz et al. (2020), Liu H.L. et al. (2018), anticancerChen et al. (2017), Gao et al. (2014), Jin et al. (2017), Kan et al. (2017), Lin et al. (2017), Marconett et al. (2010), Ozmen et al. (2010), Sun, P. et al. (2017), Zhang et al. (2017), Zhang et al. (2021), antiproliferative Kim et al. (2008), Wu and Chen, (2009), attenuates diabetic retinopathy Mei et al. (2017), neuroprotective Wu et al. (2016), improves cognition Zhang and Li, (2016), antimicrobial Sato et al. (2000), Wu, T. et al. (2015), Yu et al. (2004), acaricidal Yang et al. (2013), and antioxidant Ye and Huang, (2012)	Clears heat, resolves toxins, invigorates the blood, eliminates stasis, disperses swelling, relieves pain, and cures cancer
23	*Sphagneticola calendulacea* (L.) Pruski [*Wedelia chinensis* (Osbeck) Merr.]	*Asteraceae (Compositae)*	Huáng huā mì cài (黃花蜜菜)	0.09	Antidiabetic Chen et al. (2021), Thao et al. (2018), anticancer Huang et al. (2016), Lin et al. (2007), Liu, M. et al. (2013), Tsai et al. (2009), Tsai et al. (2015), Tsai et al. (2017a), Tsai et al. (2017b), neuroprotective Lin et al. (2014), antibacterial Darah et al. (2013), anti-inflammatory Darah et al. (2013), and antioxidant Manjamalai and Berlin Grace, (2012)	Clears heat, resolves toxins, eliminates stasis, and disperses swelling
24	*Tithonia diversifolia* (Hemsl.) A.Gray (leaf)	*Asteraceae (Compositae)*	Wǔ zhǎo jīn yīng yè(五爪金英葉)	0.09	Hepatoprotective Nguepi et al. (2021), cardioprotective Ide et al. (2020), antioxidant, antimicrobial Ferreira Farias et al. (2019), antidiabetic, immunomodulatory, analgesic, antimalarial, anti-obesity, anti-hyperlipidemic, gastroprotective, antiemetic, antidiarrheal, antileishmanial, anti-trypanosomal, antivenin, and antiviral Mabou Tagne et al. (2018)	Clears heat, resolves toxins, disperses swelling, and relieves pain

a CMUBT, china medical university bitter tea.

b No records for the stem of *T. diversifolia* in the Committee on Chinese Medicine and Pharmacy (2003) and the second edition of The Committee on Chinese Medicine and Pharmacy (2011).

### Comparison Between Taiwanese Bitter Tea and Green Herb Tea

**FIGURE 4:**
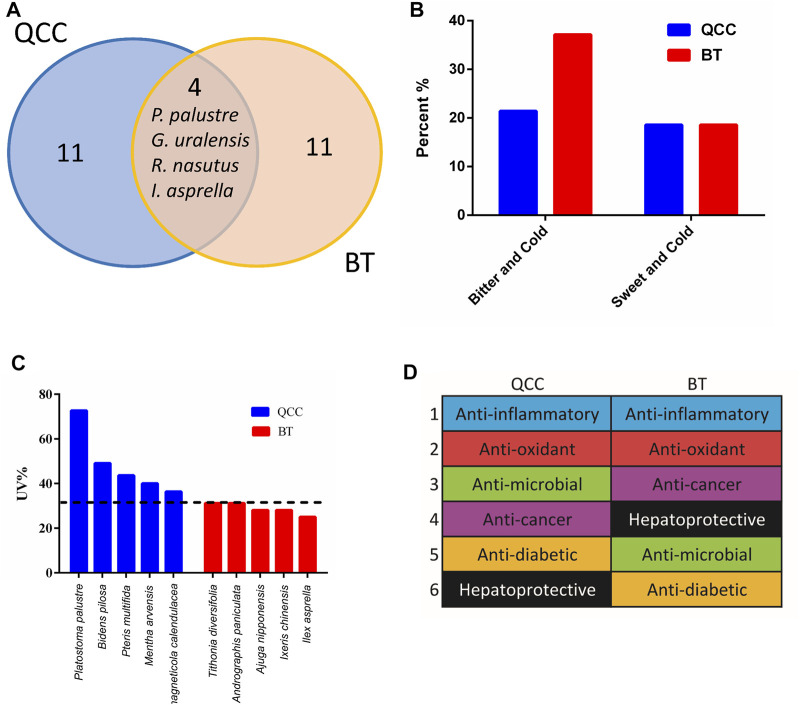
Comparisons between Taiwanese bitter tea and green herb tea. **(A)** Venn diagram of the top 15 most commonly used plant materials in bitter tea and green herb tea. **(B)** Comparison of the nature and flavor of the plant materials. **(C)** Use values of the top five most commonly used plant materials (1–5 indicate the top five commonly used plant materials). The horizontal dotted line indicates the highest UV of BT. **(D)** Modern pharmacological applications of the top 15 most commonly used plant materials. QCC, qing-cao-cha (green herb tea); BT, bitter tea.

We next compared the top 15 most commonly used plant materials between Taiwanese bitter tea and green herb tea (Figure 4A) and found an overlap of only four plant materials: *Glycyrrhiza uralensis* Fisch., *P. palustre*, *I. asprella*, and *Rhinacanthus nasutus* (L.) Kurz. Therefore, the plant materials used vary between these two tea types.

Plant materials with a bitter flavor were then divided into bitter cold and sweet cold; bitter cold plant materials accounted for 37.1 and 21.4% of the bitter tea and green herb tea plant materials, respectively, whereas sweet cold plants were used in the same proportion of approximately 19% (Figure 4B). The UV values of the top five most commonly used plant materials were higher for green herb tea than for bitter tea (Figure 4C). Moreover, the composition of green herb tea sold in different stores was shown to have minimal differences and high consistency, whereas the constituents of bitter tea differed among businesses in Taiwan. Therefore, the selection of plant materials for bitter tea is not consistent.

Anti-inflammation is the most published aspect in plant pharmacology research associated with the top 15 most commonly used plant materials in bitter tea and green herb tea (Figure 4D). For green herb tea, this is followed by antioxidant, antimicrobial, and anticancer effects, which is slightly different than that for bitter tea (Figure 4D).

### Comparison of Taiwanese Bitter Tea With That of Herbal Tea From Lingnan, Chaoshan, and Fujian

**FIGURE 5:**
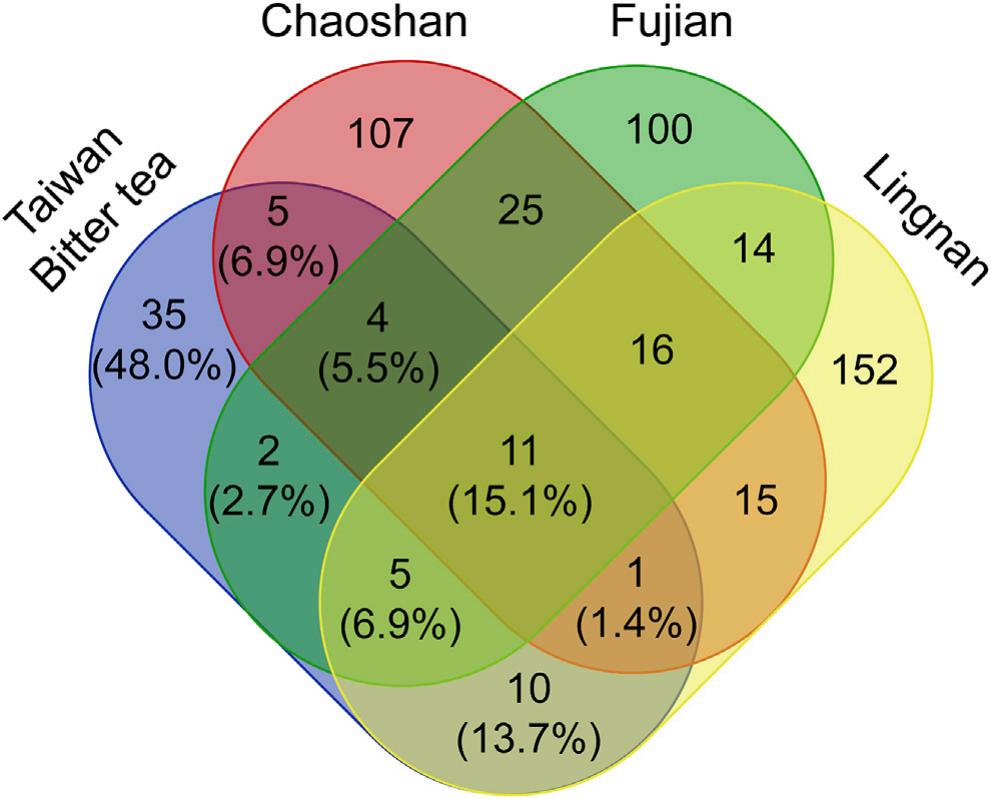
Venn diagram showing the number of shared plant materials in herbal tea from different regions.

In the south of China near Taiwan, there are other local herbal teas (liang-cha; cool tea) that are commonly consumed. Comparisons of Taiwanese bitter tea with herbal teas from the Lingnan area (Liu Y. et al., 2013), Chaoshan area (Li et al., 2017), and Fujian (Lin, 2014) revealed that the plant materials used in herbal teas vary across the four locations. Notably, 35 (48.0%) of the plant materials used in bitter tea, according to the survey, were limited to Taiwan (Figure 5).

### Analysis of High-Frequency Drug Pairs and Core Network Analysis of Taiwanese Bitter Tea

To identify the drug pairs used in high frequencies, we performed core network analysis. Eight drug pairs appeared more than four times in 32 formulations and included nine medicinal plant materials from *A. paniculata*, *I. asprella*, *T. diversifolia*, *A. nipponensis*, *Mallotus repandus* (Willd.) Muell.-Arg., *Bombax ceiba* L., *Solanum incanum* L., and *I. chinensis*. The most commonly used drug pairs were *A. paniculata*–*A. nipponensis* and *A. paniculata*–*I. asprella*, with a frequency of occurrence of 5 in 32 formulations.

**FIGURE 6:**
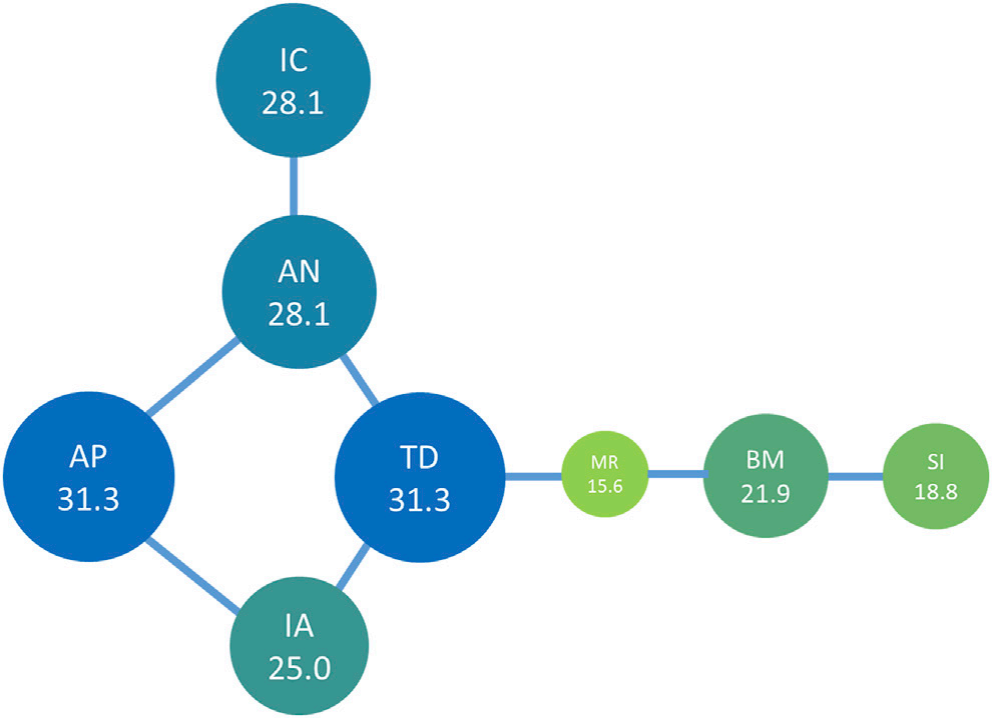
Network analysis of the high frequency drug pairs found in Taiwanese bitter tea. The number is the UV of the plant material, and the length of the connecting line indicates co-occurrence frequency. The circle sizes represent the relative ratio of UV. AN, *Ajuga nipponensis* Makino; IC, *Ixeris chinensis* (Thunb.) Nakai; IA, *Ilex asprella* (Hook. & Arn.) Champ.; TD, *Tithonia diversifolia* (Hemsl.) A. Gray; MR, *Mallotus repandus* (Willd.) Müll. Arg.; BM, *Bombax malabarica* DC.; SI, *Solanum incanum* L.; AP, *Andrographis paniculat*a (Burm. f.) Nees.

We constructed a network diagram (Figure 6) of the core components of Taiwanese bitter tea using TCMISS v2.5, and found that the core medicinal materials were from *A. nipponensis* and *T. diversifolia.* The medicinal plants often matched with *A. nipponensis* were *I. chinensis*, *A. paniculata*, and *T. diversifolia*, whereas those often matched with *T. diversifolia* were *A. nipponensis*, *I. asprella*, and *M. repandus* surrounded by *T. diversifolia*, *M. repandus*, and *S. incanum*.

## Discussion

In ethnopharmacological research, the method of field surveys have been used to explore the medicinal plants and traditional medicines prescribed for certain diseases in different regions. Topics related to medicinal botanicals that employed field investigation in the past include quality control of authentic medicinal materials (Zhao et al., 2012), green herb tea (Huang et al., 2020), herbal tea (Liu et al., 2013; Li et al., 2017), medicinal diets (Tan et al., 2017), and herbal compositions of lactation promoting herbs (Chao et al., 2020). In the present study, the field survey was adopted on Taiwanese bitter tea. We surveyed and analyzed the formulations of Taiwanese bitter tea, a special herbal tea commonly consumed in Taiwan, and revealed its current status in terms of ingredients and function.

Most of the identified plant materials in the study belonged to the family Asteraceae and Lamiaceae. The high involvement of Asteraceae and Lamiaceae in Taiwanese bitter tea is consistent with some other investigations in folk medicine outside Taiwan. They are two of the “hot families” in the medical plants (Saslis-Lagoudakis et al., 2011). Medicinal plants of the two families are dominant in Mediterranean (Gras et al., 2018), Ugandan (Tugume and Nyakoojo, 2019), Turkish (Güneş et al., 2017), Lebanese (Baydoun et al., 2015), Peruvian (Rehecho et al., 2011), Jordanian (Alzweiri et al., 2011), and Algerian (Rachid et al., 2012) folk medicines. Asteraceae exhibits high environmental adaptability and hosts a large number of naturalized and invasive plants globally. In addition, the climate in Taiwan is conducive for Asteraceae growth, and plants in this family are the third most abundant vascular plants in Taiwan, making them very easy to obtain in the wild (Rolnik and Olas, 2021). Asteraceae plants also possess numerous and diverse flavors. The Lamiaceae family is the second largest source of plant raw materials used in Taiwanese bitter tea. Lamiaceae family plants are characterized by high amounts of volatile oils, and thus the plants are extensively used as culinary herbs (Napoli et al., 2020). Previous studies have reported that the volatile oils of these plants have antioxidant, anti-inflammatory, and antibacterial properties (Nieto, 2017), which may explain why Lamiaceae is widely used in herbal tea. This study also found several naturalized plants that were introduced from America and Africa used in bitter tea. American native plants introduced into Taiwan and used in bitter tea included *Physalis angulata* L., *Bidens pilosa* L., and *Tithonia diversifolia*, and African native plants included *Solanum incanum* L. and *Momordica charantia* L. (Wu et al., 2004).

The diversity analysis for the northern, central, and southern Taiwan revealed that the more the north, the more diversity of medicinal materials were. Northern Taiwan is the political and economic center of Taiwan, with very little land to grow and correct plants. The medicinal materials used in northern Taiwan are usually imported from other areas. On the other hand, the central and southern areas of Taiwan grow more plants, including medicinal plants, and have more wild land, where wild medicinal plants could be collected. Therefore, medicinal materials used by stores in the central and southern Taiwan were more readily available and more restricted locally than those in northern Taiwan. This is the reason we speculated for the more number of medicinal materials and higher diversity in Northern Taiwan than in other areas.

All participants in the survey reported that the main function of bitter tea is to protect the liver. A previous study on green herb tea in Taiwan also reported similar results (Chen and Lin, 2012). We suspected that this result is associated with the past high prevalence of hepatitis B in Taiwan, with 15–20% of the population being chronic hepatitis B carriers. Although the Taiwanese government promoted large-scale vaccination initiatives for newborns that reduced the incidence of hepatitis B (Wait and Chen, 2012), hepatitis C—a major cause of liver cirrhosis and liver cancer—is still prevalent in Taiwan (Yu et al., 2020). According to the cause of death statistics, chronic liver disease and liver cirrhosis remain among the top 10 causes of death, with liver cancer part of the top 10 cancers in Taiwan (Ministry of Health and Welfare, 2019). Therefore, people seek local plant materials that can “protect the liver” and provide “liver protection.” Therefore, Taiwanese bitter tea may have developed as a medicinal drink under such circumstances.

Taiwanese bitter tea was mainly composed of cold and cool plant materials. According to theory of traditional Chinese medicine, cold and cool plants have a “heat clearing” effect, which can treat “heat syndrome”. Heat syndrome refers to symptoms such as redness, fever, inflammation, yellow and red urine, and dry stool (Su et al., 2013). In the survey, many storekeepers mentioned that the components rendering the bitter flavor can enter the liver and that plant raw materials of bitter plants have liver “fire reduction” effects. Therefore, Taiwanese bitter tea is used to reduce “liver fire” and improve liver health. Other commonly used traditional Chinese medicines with bitter flavor for treating liver diseases include *Gentiana scabra* Bunge, *Artemisia capillaris*, and *S. baicalensis* (Tsai et al., 2020). In addition, bitterness-imparting compounds have been extracted from *Artemisia* absinthium L., a medicinal plant with hepatoprotective effects and a strong bitter taste that has been traditionally used in Europe.

During our analysis of the use and properties of Taiwanese bitter tea plant materials in the literature, we found that hepatoprotection was the fifth most published aspect for the 24 most commonly used plant materials and the fourth for the top 15 plant materials by frequency. Plants with high UV values, such as *A. paniculate* (Hossain et al., 2014), *A. nipponensis* (Hsieh et al., 2016), and *T. diversifolia* (Mabou Tagne et al., 2018) were reported to have hepatoprotective effects in pharmacological studies, whereas *I. chinensis* was reported to have antiviral effects against the hepatitis B virus. Meanwhile, hepatoprotection was ranked sixth for Taiwanese green herb tea. The most frequently reported pharmacological action of plant materials in bitter tea was anti-inflammation, followed by anticancer, antioxidant, antimicrobial, and anti-glycosuria effects. Long-term chronic inflammation can cause excessive oxidative stress, which is a major cause of cancer development (Bishayee, 2014); therefore, anti-inflammatories and antioxidants are considered to have potential roles in preventing cancer. For Taiwanese green herb tea, the most reported pharmacological action for the plant materials was anti-inflammation, followed by antioxidant, antimicrobial, and anticancer (Huang et al., 2020). According to these data, Taiwanese green herb tea has similar benefits to those of bitter tea; however, bitter tea may have stronger hepatoprotective effects than does green herb tea.

A comparison of the composition of Taiwanese bitter tea with three popular herbal drinks from Lingnan (Liu Y. et al., 2013), Chaoshan (Li et al., 2017), and Fujian (Lin, 2014) revealed that 11 (15.1%) plants were used in common. Moreover, there are 35 (48%) plants used only in Taiwanese bitter tea but not used in the cool teas of the above-mentioned three areas of southern mainland China. Nevertheless, the functions of herbal teas advertised in these three regions in southern China are similar to those of herbal teas in Taiwan, all of which are used to “clear internal heat”. Similarly, several medicinal plants used in herbal teas in the Lingnan, Chaoshan, and Fujian were been reported to have antioxidant, anti-inflammatory (Wu et al., 2020), and hepatoprotective (Bao et al., 2008) properties in pharmacological studies. However, given that almost half of the plant materials used in Taiwanese bitter tea are not used in herbal teas from Lingnan, Chaoshan, and Fujian, Taiwanese bitter tea can be regarded as a unique herbal tea.

The store owners selling Taiwanese bitter tea had a wide age distribution. Seventy-six percent of the source of knowledge on plant materials used in Taiwanese bitter tea was information passed down over generations. Generally, the herbal tea industry is traditional and conservative, and the recipes are often restricted within families and are not shared with external parties. Such conservative practices in the tea industry are gradually being abandoned. According to a study on herbal tea culture in Taiwan, the sale of herbal tea in Taiwan has decreased, with the number of traditional stores gradually declining (Chen and Lin, 2012). The findings of the present study can facilitate the preservation of these ancient family recipes as well as the cultural heritage of Taiwanese bitter tea through the Taiwanese government, the field of ethnopharmacology, and the food and pharmaceutical industries.

## Conclusion

Bitter tea is a major traditional drink in Taiwan. The findings of the present study indicate that Taiwanese bitter tea is a very diverse mixture without a clear core species used in all these mixture. Moreover, the plant materials used in Taiwanese bitter tea are unique and that the constituent plants have numerous functions. More follow-up studies are required to ascertain the pharmacological effects of Taiwanese bitter tea; facilitate safer and more effective use of bitter tea in Taiwan; and preserve bitter tea as a traditional culture and resource.

## Data Availability

The original contributions presented in the study are included in the article/Supplementary Material, further inquiries can be directed to the corresponding authors.

## Footnotes

1
https://www.ncbi.nlm.nih.gov/pubmed

2
http://bioinformatics.psb.ugent.be/webtools/Venn/
