# Supplementary material for: Ethnobotanical Survey on Bitter Tea in Taiwan
Source: Front Pharmacol. 2022 Feb 18;13:816029. doi: 10.3389/fphar.2022.816029 (PMC8894760; doi:10.3389/fphar.2022.816029)
Supplement: Supplementary file 1 [file DataSheet1.docx]

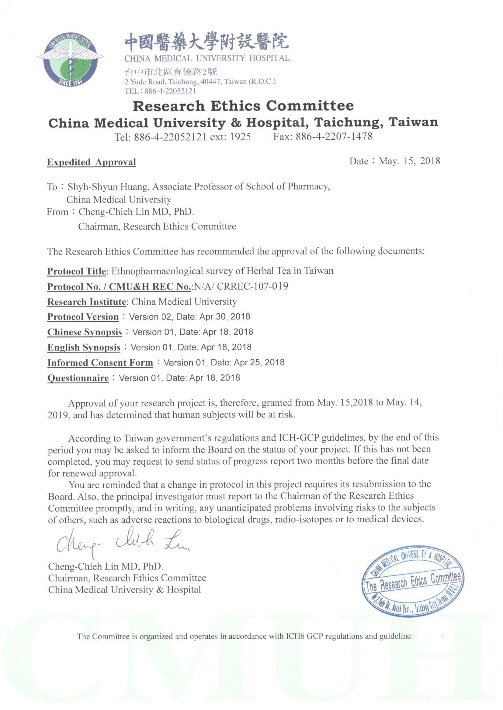


Figure S1 Approval certificate from the research ethics committee for research execution.

|   (a) |   (b) | |   (c) |
| --- | --- | --- | --- |
|   (d) | |   (e) | |
| Figure S2 Basic information of the interviewed 25 bitter tea shops. (a) Gender of the store owner; (b) Age of the store owner (years); (c) Source of the bitter tea formula of the store; (d) Operating duration of the store (years); and (e) Items sold (T: prepared tea drinks, D: dry raw material, and F: fresh raw material). | | | |
|  | | | |
|  | | | |

# Table S1 General information on plant materials of Taiwanese Bitter Tea and their traditional uses.

| No. | Scientific Name | Voucher specimen number | Family | Parts Used | Local name | Frequency | Use value (%) | Additional information | | | | Property and flavor | Traditional Use |
| --- | --- | --- | --- | --- | --- | --- | --- | --- | --- | --- | --- | --- | --- |
|  |  |  |  |  |  |  |  | Taiwan Herbal Pharmacopeia 3rd Edition | Used by China cool tea^a^ | Used by Qīng-Căo-Chá | Cultivated/Wild^d^ |  |  |
| 1 | *Adenostemma lavenia* (L.) Kuntze | CMUBT-38 | Asteraceae (Compositae) | Whole plant | Má shǔ hú  (麻糬糊) | 1 | 0.03 |  |  |  | C/W | Pungent and sweet flavor, and slightly cool in nature. | Clear heat, benefit urination, and remove toxins for detumescence. |
| 2 | *Ajuga nipponensis* Makino | CMUBT-3 | Lamiaceae | Whole plant | Bái mǎ wú gōng  (白馬蜈蚣) | 9 | 0.28 |  |  | Y | C/W | Bitter flavor and cool in nature. | Alleviate inflammation, cool blood, and help in bone connection. |
| 3 | *Alternanthera sessilis* (L.) R.Br. ex DC. | CMUBT-39 | Amaranthaceae | Whole plant | Hóng tián wū  (紅田烏) | 1 | 0.03 |  | BC | Y | C/W | Bitter flavor and cool in nature. | Clear heat, benefit urination, and eliminate toxins. |
| 4 | *Andrographis paniculata* (Burm. f.) Nees | CMUBT-1 | Acanthaceae | Whole plant | Chuān xīn lián  (穿心蓮) | 10 | 0.31 |  |  |  | C/W | Bitter flavor, cool in nature, and poisonous. | Clear heat and toxins, and reduce swelling to relieve pain. |
| 5 | *Angelica keiskei* Koidzumi | CMUBT-40 | Umbelliferae | Aerial parts | Míng rì yè  (明日葉) | 1 | 0.03 |  |  |  | C | None | Clear heat, benefit urination, strengthen the body, and promote lactation. |
| 6 | *Artemisia capillaris* Thunb^c^ | CMUBT-25 | Asteraceae (Compositae) | Whole plant | Yīn chén  (茵陳) | 2 | 0.06 |  |  | Y | W | Bitter flavor and cool in nature. | Clear heat, and benefit urination and bile-draining to relieve jaundice symptoms. |
| 7 | *Astragalus propinquus* Schischkin^c^  [*Astragalus membranaceus* (Fisch.) Bunge var. *mongholicus* (Bunge) P.K.Hsiao]^b^ | CMUBT-41 | Leguminosae | Root | Huáng qí  (黃耆) | 1 | 0.03 |  |  | Y | － | Sweet flavor and warm in nature. | Tonify qi and secure the exterior. Benefit urination and remove toxins and pus. Treat sores and develop flesh. |
| 8 | *Bidens pilosa* L.  [*Bidens pilosa* L. var. *radiata* Sch. Bip.] | CMUBT-16 | Asteraceae (Compositae) | Whole plant | Xián fēng cǎo  (咸豐草) | 3 | 0.09 |  | B | Y | C/W | Sweet and slightly bitter flavor and neutral in nature. | Clear heat and benefit urination. Promote bile-draining to relieve jaundice symptoms. |
| 9 | *Boehmeria nivea* (L.) Gaudich.  [*Boehmeria nivea* (L.) Gaudich. var. *tenacissima* (Gaudich.) Miq.] | CMUBT-12 | Urticaceae | Stem | Shān zhù má  (山苧麻) | 4 | 0.13 |  |  | Y | W | Sweet flavor and cool in nature. | Alleviate inflammation and clear heat. |
| 10 | *Bombax ceiba* L.  [*Bombax malabaricum* DC.] | CMUBT-6 | Malvaceae  [Bombacaceae] | Root | Mù mián gēn  (木棉根) | 7 | 0.22 |  |  | Y | C | Pungent flavor and neutral in nature. | Eliminate wind and remove dampness. Cool and remove toxins. Alleviate static blood and relieve pain. |
| 11 | *Bupleurum chinense* DC. | CMUBT-42 | Apiaceae | Root | Chái hú  (柴胡) | 1 | 0.03 |  | A |  | － | Bitter and pungent flavor and slightly cold nature. | Relieve the external parts to abate heat; disperse stagnated liver qi to relieve qi stagnation, and uplift the qi of yang. |
| 12 | *Centella asiatica* (L.) Urb. | CMUBT-43 | Apiaceae | Whole plant | Hán ké cǎo  (含殼草) | 1 | 0.03 |  | ABC | Y | C/W | Bitter and pungent flavor. Cold and slightly poisonous in nature. | Alleviate inflammation and emove toxins. Cool blood and promote fluid production. Clear heat and benefit urination. |
| 13 | *Cirsium brevicaule* A.Gray | CMUBT-44 | Asteraceae (Compositae) | Whole plant | Jī jiǎo cì  (雞角刺) | 1 | 0.03 |  |  |  | C/W | Sweet flavor and cool in nature. | Cool blood and promote blood circulation. Remove blood stasis to alleviate edema. Benefit urination and supplement vacuity. |
| 14 | *Citrus reticulata* Blanco | CMUBT-45 | Rutaceae | Peel | Chén pí  (陳皮) | 1 | 0.03 |  | A |  | C | Pungent and bitter flavor and warm in nature. | Activate qi and invigorate the spleen. Lower adverse qi and treat phlegm. |
| 15 | *Codonopsis pilosula* (Franch.) Nannf. | CMUBT-46 | Campanulaceae | Root | Dǎng sān  (黨參) | 1 | 0.03 |  | A |  | C | Sweet flavor and neutral in nature. | Invigorate the spleen and lungs. Benefit qi and promote fluid production. |
| 16 | *Coptis chinensis* Franch. | CMUBT-47 | Ranunculaceae | rhizome | Huáng lián  (黃連) | 1 | 0.03 |  | A |  | － | Bitter flavor and cool in nature. | Clear heat and relieve fire and dry dampness. Remove toxins. |
| 17 | *Crataegus pinnatifida* Bunge | CMUBT-48 | Rosaceae | Fruit | Shān zhā  (山楂) | 1 | 0.03 |  | A |  | － | Sour and sweet flavor and slightly warm and non-toxic in nature. | Disperse food and treat stasis. |
| 18 | *Curcuma aromatica* Salisb.  [*Curcuma wenyujin* Y.H.Chen et C.Ling] | CMUBT-49 | Zingiberaceae | Root | Yù jīn  (鬱金) | 1 | 0.03 |  |  |  | C | Pungent and bitter flavor and cool in nature. | Promote blood circulation and stop pain. Promote qi to relieve depression. Clean the heart and cool blood. Soothe the liver to benefit the bile. |
| 19 | *Curcuma longa* L. | CMUBT-50 | Zingiberaceae | Rhizome | Jiāng huáng  (薑黃) | 1 | 0.03 |  |  |  | C | Pungent and bitter flavor and warn in nature. | Anticancertreatment. Promote blood circulation to treat blood stasis. Disperse stasis to treat static blood. |
| 20 | *Elephantopus scaber* L. | CMUBT-17 | Asteraceae (Compositae) | Whole plant | Ding shù wū  (丁豎杇) | 3 | 0.09 |  | ABC | Y | C/W | Bitter flavor and cold in nature. | Clear heat and toxins. Benefit urination and alleviate edema. |
| 21 | *Eleutherococcus senticosus* (Rupr. & Maxim.) Maxim.  [*Acanthopanax senticosus* (Rupr. et Maxim.) Harms] | CMUBT-51 | Araliaceae | Stem | Cì wǔ jiā  (刺五加) | 1 | 0.03 |  |  | Y | C | Bitter and pungent flavor and warm in nature. | Invigorate the kidney and strengthen the waist. Replenish qi and soothe the nerves. Promote blood circulation and dredge collaterals. |
| 22 | *Ganoderma lucidum* (Leyss. ex Fr.) Karst. | CMUBT-52 | Polyporaceae | Fruiting body | Líng zhī  (靈芝) | 1 | 0.03 |  |  |  | C/W | Sweet and slightly bitter flavor and neutral in nature. | Replenish qi and blood. Nourish the heart and calm the nerves. Relieve cough and calm panting. |
| 23 | *Gardenia jasminoides* J.Ellis | CMUBT-53 | Rubiaceae | Root | Zhī zi gēn  (梔子根) | 1 | 0.03 |  |  |  | C/W | Sweet and bitter flavor and cold in nature. | Clear heat and dampness. Cool blood to stop bleeding. |
| 24 | *Glossocardia bidens* (Retz.) Veldkamp | CMUBT-54 | Asteraceae (Compositae) | Whole plant | Fēng rú cǎo  (風茹草) | 1 | 0.03 |  |  | Y | C/W | Slightly pungent, sweet, and slightly bitter flavor and cold in nature. | Clear heat and toxins. Remove dampness and swelling. Promote blood circulation to remove blood stasis. |
| 25 | *Glycyrrhiza uralensis* Fisch. | CMUBT-13 | Leguminosae | Root & rhizome | Gān cǎo  (甘草) | 4 | 0.13 |  | A | Y | － | Sweet flavor and neutral in nature. | Replenish qi and invigorate the spleen. Relax tension and relieve pain. Hydrate the lungs to relieve cough. Relieve fire and remove toxins. Reconcile various medicines. |
| 26 | *Gynostemma pentaphyllum* (Thunb.) Makino | CMUBT-26 | Cucurbitaceae | Whole plant | Qī yè dǎn  (七葉膽) | 2 | 0.06 |  | AC |  | C/W | Slightly sweet and bitter flavor and cool in nature. | Clear heat and toxins. Relieve cough and expel phlegm. Supplement any deficiencies. |
| 27 | *Ilex asprella* (Hook. & Arn.) Champ. ex Benth. | CMUBT-5 | Aquifoliaceae | Stem & Root | Wàn diǎn jīn  (萬點金) | 8 | 0.25 |  | AC | Y | W | Sweet flavor and cold in nature. | Cool blood to stop bleeding. Clear heat and benefit urination. |
| 28 | *Imperata cylindrica* (L.) Raeusch.^c^  [*Imperata cylindrica* (L.) P. Beauv. var. *major* (Nees) C. E. Hubb.] | CMUBT-55 | Poaceae | Rhizome | Bái máo gēn  (白茅根) | 1 | 0.03 |  | ABC | Y | W | Bitter flavor and cool in nature. | Clear heat and toxins. Purge fire and cool blood to stop bleeding. Relieve pain, regulate menstruation, and promote blood circulation. Dispel putridity and develop flesh.  Clear heat, benefit urination, and soothe the nerves. |
| 29 | *Ixeris chinensis* (Thunb. ex Thunb.) Nakai | CMUBT-4 | Asteraceae (Compositae) | Whole plant | Xiǎo jīn yīng  (小金英) | 9 | 0.28 |  |  | Y | C/W | Slightly sweet flavor and cold in nature. | Clear heat, benefit urination, and soothe the nerves. |
| 30 | *Juncus effusus* L. | CMUBT-56 | Juncaceae | Pith | Dēng xīn cǎo  (燈心草) | 1 | 0.03 |  | ABC |  | W | Pungent, astringent, and bitter flavor, and neutral in nature. | Clear heat, quench thirst, relieve pain, disperse wind, relax sinews, cool blood to stop dysentery, reduce swelling, and promote blood circulation. |
| 31 | *Kadsura japonica* (L.) Dunal | CMUBT-57 | Schisandraceae  [Magnoliaceae] | Stem | Hóng gǔ shé  (紅骨蛇) | 1 | 0.03 |  |  | Y | W | Sweet flavor cool in nature. | Clear heat and remove toxins. Treat sore throat, influenza, tonsillitis, acute mastitis, intestinal abscess, furuncles, and carbuncles, pus and swelling, erysipelas, traumatic infection, and vaginal discharge. |
| 32 | *Lonicera japonica* Thunb. | CMUBT-58 | Caprifoliaceae | Bud | Jīn yín huā  (金銀花) | 1 | 0.03 |  | ABC |  | C/W | Bitter flavor and cold in nature. | Clear heat and remove toxins. Benefit urination, and stop bleeing. |
| 33 | *Ludwigia octovalvis* (Jacq.) P.H.Raven | CMUBT-59 | Onagraceae | Root | Shuǐ dīng xiāng  (水丁香) | 1 | 0.03 |  |  |  | W | Sweet and slightly bitter flavor and cold in nature. | Disperse wind and dampness. Promote blood circulation and dredge collaterals. Remove toxins and reduce swelling. Expel worms and relieve itching. |
| 34 | *Mallotus repandus* (Willd.) Muell.-Arg. | CMUBT-8 | Euphorbiaceae | Stem | Tǒng jiāo téng  (桶交藤) | 5 | 0.16 |  |  | Y | W | Pungent flavor. It is cool in nature is and has an aroma. | Clear heat and relieve the external parts. Sooth the throat and outthrust rashes. Invigorate the stomach, reduce swelling, and relieve itching. |
| 35 | *Mentha arvensis* L. | CMUBT-27 | Lamiaceae | Whole plant | Bó hé  (薄荷) | 2 | 0.06 |  | ABC | Y | C/W | Sweet flavor and cool in nature. | Clear heat, quench thirst, cool blood, release summer heat, and lower blood pressure. |
| 36 | *Momordica charantia* L.  [*Momordica charantia* L. var. *abbreviata* Ser.] | CMUBT-18 | Cucurbitaceae | Stem | Shān kǔ guā  (山苦瓜) | 3 | 0.09 |  |  |  | W | Bitter and sweet flavor and cold in nature. | Disperse the wind and clear heat. Remove liver-fire for improving eyesight. |
| 37 | *Morus alba* L. | CMUBT-28 | Moraceae | Leaf | Sāng yè  (桑葉) | 2 | 0.06 |  | ABC | Y | C/W | Bitter and astringent flavor and slightly warm in nature. | Relax sinews and activate collaterals. Tonify blood and promote blood circulation. Clear lungs and hydrate dryness. Regulate menstruation. |
| 38 | *Mucuna macrocarpa* Wall. | CMUBT-19 | Leguminosae | Stem | Xiě téng  (血藤) | 3 | 0.09 |  |  | Y | W | Pungent flavor and warm in nature. | Treat rheumatic backache. |
| 39 | *Ocimum gratissimum* L. | CMUBT-29 | Lamiaceae | Stem | Shān jiǔ céng tǎ  (山九層塔) | 2 | 0.06 |  | B | Y | C | Bitter and sweet flavor and cold in nature. | Clear heat and remove toxins. Remove dampness and carbuncle. Fight cancer. |
| 40 | *Odontosoria chinensis* (L.) J. Sm.  [*Sphenomeris chusana* (L.) Copel.] | CMUBT-30 | Lindsaeaceae | Whole plant | Shuǐ jī zhǎo  (水雞爪) | 2 | 0.06 |  |  |  | W | Slightly bitter and astringent flavor and cold in nature. | Clear heat and benefit urination. Stop bleeding and develop flesh. Alleviate inflammation, remove toxins, and clear heart fire. |
| 41 | *Oldenlandia diffusa* (Willd.) Roxb.^c^  [*Hedyotis diffusa* Willd.] | CMUBT-20 | Rubiaceae | Whole plant | Bái huā shé shé cǎo  (白花蛇舌草) | 3 | 0.09 |  | ABC | Y | W | Bitter and sweet flavor and cold in nature. | Clear heat and toxins. Promote body fluid production to quench thirst. Promote blood circulation. |
| 42 | *Onychium japonicum* (Thunb.) Kunze | CMUBT-60 | Pteridaceae | Whole plant | Fèng wěi lián  (鳳尾連) | 1 | 0.03 |  |  | Y | W | Bitter flavor and cold in nature. | Clear heat, dampnes, and toxins, and stop bleeding. It is a bitter stomachic. |
| 43 | *Ophioglossum petiolatum* Hook. | CMUBT-61 | Ophioglossaceae | Whole plant | Yī yè cǎo  (一葉草) | 1 | 0.03 |  |  |  | W | Bitter and sweet flavor and cold in nature. | Clear heat and remove toxins. Reduce swelling and relieve pain. |
| 44 | *Orthosiphon aristatus* (Blume) Miq. | CMUBT-14 | Lamiaceae | Whole plant | Huà shí cǎo  (化石草) | 4 | 0.13 |  | BC | Y | C | Sweet and slightly bitter flavor and cool in nature. | Clear heat, benefit urination, and expel stones. |
| 45 | *Perilla frutescens* (L.) Britton | CMUBT-62 | Lamiaceae | Leaf | Zǐ sū  (紫蘇) | 1 | 0.03 |  | AB | Y | C | Pungent flavor and warm in nature. | Relieve the external parts and dispel cold. Regulate qi and disperse food. Promote qi and harmonize the stomach. |
| 46 | *Phellodendron chinense* C.K.Schneid. | CMUBT-31 | Rutaceae | Bark | Huáng bó  (黃柏) | 2 | 0.06 |  |  |  | － | Bitter flavor cold in nature. | Clear heat and dry dampness. Purge fire and remove toxin. |
| 47 | *Phyllanthus urinaria* L. | CMUBT-63 | Phyllanthaceae | Whole plant | Yè xià zhū  (葉下珠) | 1 | 0.03 |  | BC |  | W | Sweet and bitter flavor and cool in nature. | Clear heat and benefit urination. Disperse accumulation and brighten the eyes. Diminish inflammation. Pacify the liver and remove toxins. |
| 48 | *Physalis angulata* L. | CMUBT-9 | Solanaceae | Whole plant | Dēng long cǎo  (燈籠草) | 5 | 0.16 |  | B |  | W | Sour and bitter flavor and cold in nature. | Clear heat and remove toxins. Reduce swelling and disperse stasis. |
| 49 | *Plantago asiatica* L. | CMUBT-64 | Plantaginaceae | Whole plant | Chē qián cǎo  (車前草) | 1 | 0.03 |  | ABC | Y | C/W | Sweet flavor cold in nature. | Clear heat and benefit urination. Expel phlegm. Cool blood and remove the toxins. |
| 50 | *Platostoma palustre* (Blume) A.J.Paton  [*Mesona chinensis* Benth.] | CMUBT-15 | Lamiaceae | Whole plant | Xiān cǎo  (仙草) | 4 | 0.13 |  | AC | Y | C | Bitter flavor and cold in nature. | Clear heat and remove toxins. |
| 51 | *Pogonatherum crinitum* (Thunb.) Kunth | CMUBT-65 | Poaceae | Whole plant | Bǐ zī cǎo  (筆仔草) | 1 | 0.03 |  | ABC | Y | C/W | Sweet flavor and cold in nature. | Clear heat and remove toxins. Dry dampness and free strangury. Cool blood, and fight cancer. |
| 52 | *Prunella vulgaris* L. | CMUBT-66 | Lamiaceae | Spike | Xià kū cǎo  (夏枯草) | 1 | 0.03 |  | ABC | Y | C/W | Bitter and pungent flavor and cold in nature. | Clear the liver, dispel stasis, and reduce swelling. |
| 53 | *Pteris multifida* Poir. | CMUBT-21 | Pteridaceae | Whole plant | Fèng wěi cǎo  (鳳尾草) | 3 | 0.09 |  | C | Y | C/W | Bitter flavor and slightly cold in nature. | Clear heat and dampness. Cool blood and remove toxins. |
| 54 | *Rheum palmatum* L. | CMUBT-67 | Polygonaceae | Rhizome | Dà huáng  (大黃) | 1 | 0.03 |  | A |  | － | Bitter flavor and cold in nature. | Remove stagnation, clear dampness-heat. Purge fire, cool blood, remove blood stasis and toxins. |
| 55 | *Rhinacanthus nasutus* (L.) Kurz | CMUBT-10 | Acanthaceae | Whole plant | Bái hè líng zhī  (白鶴靈芝) | 5 | 0.16 |  | B | Y | C | Sweet and slightly bitter flavor and neutral and slightly toxic in nature. | Nourish the lungs and relieve cough. Pacify the liver and alleviate fire. Reduce swelling and remove toxins. Kill insects and alleviate itching. |
| 56 | *Salvia miltiorrhiza* Bunge | CMUBT-68 | Lamiaceae | Root & rhizome | Dān shēn  (丹參) | 1 | 0.03 |  | A |  | C | Bitter flavor and slightly cold in nature. | Promote blood circulation and remove blood stasis. Tranquilize and calm the heart. Regulate menstruation and relieve pain. Cool blood and reduce swelling. |
| 57 | *Salvia plebeia* R. Br. | CMUBT-32 | Lamiaceae | Whole plant | Qī céng tǎ  (七層塔) | 2 | 0.06 |  |  | Y | C/W | Bitter and pungent flavor and cool in nature. | Clear heat and remove toxins, and benefit urination. |
| 58 | *Schisandra chinensis* (Turcz.) Baill. | CMUBT-69 | Schisandraceae  [Magnoliaceae] | Fruit | Wǔ wèi zǐ  (五味子) | 1 | 0.03 |  | A |  | － | Sour flavor and warm in nature. | Promote astringency. Nourish qi and promote fluid production. Calm and tranquilize the heart. |
| 59 | *Scutellaria baicalensis* Georgi | CMUBT-33 | Lamiaceae | Root | Huáng qín  (黃芩) | 2 | 0.06 |  | A |  | － | Bitter flavor and cold in nature. | Clear heat and dry dampness. Purge fire and remove toxins. Stop bleeding and prevent abortion. |
| 60 | *Scutellaria barbata* D. Don | CMUBT-22 | Lamiaceae | Whole plant | Bàn zhī lián  (半枝蓮) | 3 | 0.09 |  | BC | Y | C/W | Pungent flavor and neutral in nature. | Clear heat and remove toxins. Promote blood circulation and remove blood stasis. Reduce swelling and relieve pain. Fight cancer. |
| 61 | *Senna tora* (L.) Roxb.  [*Cassia tora* L.] | CMUBT-70 | Leguminosae | Seed | Jué míng zǐ  (決明子) | 1 | 0.03 |  | AC |  | C/W | Bitter, sweet, and salty flavor and cool in nature. | Laxation to relax the bowels. Clear the liver and improve eyesight. Benefit urination, and relax the bowels. |
| 62 | *Sigesbeckia orientalis* L. | CMUBT-11 | Asteraceae (Compositae) | Aerial parts | Kǔ cǎo  (苦草)  Xī liàn cǎo  (豨薟草) | 5 | 0.16 |  |  | Y | W | Bitter flavor and cold in nature. | Eliminate rheumatismand benefit the bones and muscles. |
| 63 | *Solanum americanum* Mill.  [*Solanum nigrum* L.] | CMUBT-71 | Solanaceae | Whole plant | Lóng kuí  (龍葵) | 1 | 0.03 |  |  | Y | W | Bitter and slightly sweet flavor. Cold in nature with slight toxicity. | Clear heat and remove toxins. Reduce swelling and dispel stasis. Promote blood circulation and diuresis. |
| 64 | *Solanum incanum* L. | CMUBT-7 | Solanaceae | Stem | Huáng shuǐ qié  (黃水茄) | 6 | 0.19 |  |  | Y | C | Bitter flavor and cool in nature with toxicity. | Reduce inflammation and remove toxins. Disperse wind and relieve pain. Clear heat and diminish inflammation. |
| 65 | *Solanum violaceum* Ortega | CMUBT-34 | Solanaceae | Stem | Liǔ zī qié  (柳仔茄) | 2 | 0.06 |  |  | Y | C | Bitter flavor and neutral in nature with slight toxicity. | Clear heat and dampness. Remove blood stasis and reduce swelling. |
| 66 | *Sophora flavescens* Aiton | CMUBT-35 | Leguminosae | Root | Kǔ shēn gēn  (苦參根) | 2 | 0.06 |  | C |  | － | Bitter Flavor and cold in nature. | Clear heat and dry dampness. Replenish wind and kill insects. Benefit urination. |
| 67 | *Sphagneticola calendulacea* (L.) Pruski  [*Wedelia chinensis* (Osbeck) Merr.] | CMUBT-23 | Asteraceae (Compositae) | Whole plant | Huáng huā mì cài  (黃花蜜菜) | 3 | 0.09 |  | B | Y | C/W | Sweet flavor and cool in nature. | Clear heat and remove toxins. Remove blood stasis and reduce swelling. |
| 68 | *Stevia rebaudiana* (Bertoni) Bertoni | CMUBT-72 | Asteraceae (Compositae) | Leaf | Tián jú  (甜菊) | 1 | 0.03 |  |  | Y | C | Sweet flavor and neutral in nature. | Strengthen the body, harmonize the stomach, control birth, promote body fluid production, and relieve cough, and lower blood pressure. |
| 69 | *Taraxacum campylodes* G.E.Haglund^c^  [*Taraxacum officinale* Weber ex Wiggers] | CMUBT-36 | Asteraceae (Compositae) | Whole plant | Pú gōng yīng  (蒲公英) | 2 | 0.06 |  | AC | Y | C/W | Bitter and sweet flavor and cold in nature. | Clear heat and remove toxins. Benefit urination, eliminate carbuncle and disperse stasis. |
| 70 | *Terminalia catappa* L. | CMUBT-37 | Combretaceae | Leaf | Lǎn rén yè  (欖仁葉) | 2 | 0.06 |  |  |  | C | Pungent and slightly bitter flavor and cool in nature. | Disperse wind and clear heat. Relieve cough and pain. Remove toxins and kill insects. |
| 71 | *Tithonia diversifolia* (Hemsl.) A.Gray | CMUBT-2 | Asteraceae (Compositae) | Stem | Wǔ zhǎo jīn yīng  (五爪金英) | 10 | 0.31 |  |  | Y | C/W | None | None |
| 72 |  | CMUBT-24 |  | Leaf | Wǔ zhǎo jīn yīng yè  (五爪金英葉) | 3 | 0.09 |  |  |  | C/W | Bitter flavor and cool in nature with toxicity. | Clear heat and remove toxins. Reduce swelling and relieve pain. |
| 73 | *Uncaria hirsuta* Havil. | CMUBT-73 | Rubiaceae | Stem | Dào diào fēng  (倒吊風) | 1 | 0.03 |  |  |  | W | Sweet flavor and cool in nature. | Clear heat and pacify the liver. Extinguish the wind and relieve convulsions. |

^a^Regional comparison code: A, Lingnan; B, Chaoshan; C Fujian

^b^If [genus species] appears in the scientific name column, it indicates that the scientific name in the box is inconsistent with “The Plant List,” which is the common scientific name in the third edition of the “Taiwan Herbal Pharmacopeia,” second edition of “Flora of Taiwan,” or the Taiwan Biodiversity Information Facility Portal (http://taibif.tw/).

^c^Indicates that other plants may be mixed with medicinal materials during their use: Example, *Artemisia capillaris* Thunb. may be mixed with *Origanum vulgare* L., *Astragalus propinquus* Schischkin, and *Hedysarum polybotrys* Hand.-Mazz.; *Imperata cylindrica* (L.) Raeusch. may be mixed with *Pennisetum flaccidum* Griseb.; *Oldenlandia diffusa* (Willd.) Roxb. may be mixed with *Oldenlandia corymbosa* L.; and *Taraxacum campylodes* G.E.Haglund may be mixed wiyh *Ixeris chinensis* (Thunb.) Nakai.

^d^Cultivated/Wild: C, Culvated; W, Wild; －, none of the above.

| **Table S2 The recipes of medicinal materials used by Taiwanese bitter tea stores.** | | | | | | | | | | | | | | | | | | | | | | | | | | | | | | | | |
| --- | --- | --- | --- | --- | --- | --- | --- | --- | --- | --- | --- | --- | --- | --- | --- | --- | --- | --- | --- | --- | --- | --- | --- | --- | --- | --- | --- | --- | --- | --- | --- | --- |
| Bitter tea store | N1^a^ | N2 | N3 | N4 | N5 | N6 | N7 | N8 | N9 | N10 | N11 | N12 | N13 | N14 | N15 | C1^b^ | C2 | C3 | C4 | C5 | C6 | C7 | C8 | C9 | S1^c^ | S2 | S3 | S4 | S5 | S6 | E1^d^ | E2 |
| Tithonia diversifolia (stem) | 1 | 1 |  | 1 |  | 1 | 1 |  | 1 |  |  | 1 |  |  |  | 1 |  |  |  |  |  |  |  |  | 1 |  |  |  | 1 |  |  |  |
| Andrographis paniculata | 1 |  | 1 |  |  | 1 |  |  |  | 1 |  |  | 1 | 1 | 1 |  | 1 |  | 1 |  |  |  |  |  | 1 |  |  |  |  |  |  |  |
| Ixeris chinensis |  |  |  |  |  |  | 1 | 1 |  | 1 |  |  |  | 1 |  |  | 1 |  |  |  | 1 |  |  |  |  |  |  | 1 | 1 |  | 1 |  |
| Ajuga nipponensis | 1 |  |  |  |  | 1 | 1 | 1 |  |  |  |  |  | 1 |  | 1 | 1 |  | 1 |  |  |  |  | 1 |  |  |  |  |  |  |  |  |
| Ilex asprella | 1 |  |  | 1 |  | 1 |  |  | 1 | 1 |  |  | 1 |  |  |  |  |  | 1 |  |  |  | 1 |  |  |  |  |  |  |  |  |  |
| Bombax ceiba | 1 | 1 |  |  | 1 |  |  |  |  |  |  |  |  |  |  | 1 |  | 1 |  | 1 |  |  | 1 |  |  |  |  |  |  |  |  |  |
| Solanum incanum | 1 | 1 |  |  | 1 |  |  |  |  |  | 1 |  |  |  |  |  |  |  |  | 1 |  |  |  |  |  | 1 |  |  |  |  |  |  |
| Rhinacanthus nasutus |  |  |  |  |  | 1 |  |  |  |  | 1 |  |  |  |  |  |  |  |  |  | 1 |  |  |  | 1 | 1 |  |  |  |  |  |  |
| Mallotus repandus | 1 | 1 |  |  |  |  |  |  |  |  |  |  |  |  |  | 1 |  |  |  | 1 |  |  |  |  |  |  |  |  | 1 |  |  |  |
| Physalis angulata |  |  | 1 |  |  |  |  |  |  |  |  |  |  |  |  | 1 | 1 |  |  |  |  |  |  |  |  |  |  |  |  |  | 1 | 1 |
| Sigesbeckia orientalis | 1 |  |  |  | 1 | 1 |  |  |  |  |  |  | 1 |  |  |  |  |  |  |  |  |  |  |  |  |  |  |  |  |  |  | 1 |
| Orthosiphon aristatus |  |  | 1 |  |  |  |  |  |  |  |  |  |  |  |  |  |  |  |  | 1 |  |  |  |  | 1 | 1 |  |  |  |  |  |  |
| Platostoma palustre |  |  |  |  |  |  |  |  |  |  |  |  |  |  |  | 1 |  |  |  |  |  |  |  |  | 1 | 1 |  |  |  | 1 |  |  |
| Glycyrrhiza uralensis |  |  |  |  |  |  |  |  |  |  | 1 |  | 1 |  |  |  |  |  |  |  |  |  |  |  |  |  | 1 |  |  | 1 |  |  |
| Boehmeria nivea | 1 | 1 |  |  |  |  |  |  |  |  |  |  |  |  |  |  |  |  |  | 1 |  |  | 1 |  |  |  |  |  |  |  |  |  |
| Momordica charantia |  | 1 |  |  | 1 |  |  |  |  |  |  |  |  |  |  |  |  |  |  |  |  |  |  |  |  |  |  |  | 1 |  |  |  |
| Tithonia diversifolia (leaf) |  |  |  |  | 1 |  |  | 1 |  |  |  |  | 1 |  |  |  |  |  |  |  |  |  |  |  |  |  |  |  |  |  |  |  |
| Scutellaria barbata |  |  |  |  |  |  |  |  |  |  |  | 1 |  |  |  | 1 | 1 |  |  |  |  |  |  |  |  |  |  |  |  |  |  |  |
| Oldenlandia diffusa |  |  |  |  |  |  |  |  |  |  |  | 1 |  |  |  | 1 | 1 |  |  |  |  |  |  |  |  |  |  |  |  |  |  |  |
| Mucuna macrocarpa |  |  |  |  | 1 |  |  |  |  |  |  | 1 |  |  |  | 1 |  |  |  |  |  |  |  |  |  |  |  |  |  |  |  |  |
| Bidens pilosa |  |  |  |  |  |  |  |  |  |  |  |  |  |  |  | 1 |  |  |  |  | 1 |  |  |  |  | 1 |  |  |  |  |  |  |
| Sphagneticola calendulacea |  |  |  |  |  | 1 |  |  |  |  |  |  |  |  |  | 1 |  |  |  |  |  |  |  |  |  | 1 |  |  |  |  |  |  |
| Pteris multifida |  |  |  |  |  | 1 |  |  |  |  |  |  |  |  |  | 1 |  |  |  |  |  |  |  |  |  |  |  |  |  | 1 |  |  |
| Elephantopus scaber |  | 1 |  |  |  |  |  |  |  |  |  |  |  |  |  |  |  | 1 |  |  |  |  |  |  |  |  |  |  |  |  |  |  |
| Gynostemma pentaphyllum |  |  |  |  |  |  |  |  |  | 1 |  |  |  | 1 |  |  |  |  |  |  |  |  |  |  |  |  |  |  |  |  |  |  |
| Ocimum gratissimum | 1 | 1 |  |  |  |  |  |  |  |  |  |  |  |  |  |  |  |  |  |  |  |  |  |  |  |  |  |  |  |  |  |  |
| Salvia plebeia |  |  |  | 1 |  |  |  |  |  |  |  |  |  |  |  | 1 |  |  |  |  |  |  |  |  |  |  |  |  |  |  |  |  |
| Odontosoria chinensis | 1 | 1 |  |  |  |  |  |  |  |  |  |  |  |  |  |  |  |  |  |  |  |  |  |  |  |  |  |  |  |  |  |  |
| Solanum violaceum | 1 | 1 |  |  |  |  |  |  |  |  |  |  |  |  |  |  |  |  |  |  |  |  |  |  |  |  |  |  |  |  |  |  |
| Sophora flavescens |  |  |  |  |  |  |  |  |  |  |  |  |  |  |  |  |  |  |  |  |  |  |  |  |  |  | 1 |  |  |  | 1 |  |
| Morus alba |  | 1 |  |  |  |  |  |  |  |  |  |  |  |  |  |  |  |  |  |  |  |  |  |  |  |  |  |  |  | 1 |  |  |
| Artemisia capillaris |  |  |  |  | 1 |  |  |  |  |  | 1 |  |  |  |  |  |  |  |  |  |  |  |  |  |  |  |  |  |  |  |  |  |
| *Scutellaria baicalensis* |  |  |  |  |  |  |  |  |  |  |  |  |  |  |  |  |  |  |  |  |  |  |  |  |  |  |  |  |  |  | 1 | 1 |
| Phellodendron chinense |  |  |  |  |  |  |  |  |  |  |  |  |  |  |  |  |  |  |  |  |  |  |  |  |  |  | 1 |  |  |  |  | 1 |
| Taraxacum campylodes | 1 | 1 |  |  |  |  |  |  |  |  |  |  |  |  |  |  |  |  |  |  |  |  |  |  |  |  |  |  |  |  |  |  |
| Mentha arvensis |  |  |  |  |  |  |  |  |  |  | 1 |  |  |  |  |  |  |  |  |  |  |  |  |  |  |  |  |  |  | 1 |  |  |
| Terminalia catappa | 1 | 1 |  |  |  |  |  |  |  |  |  |  |  |  |  |  |  |  |  |  |  |  |  |  |  |  |  |  |  |  |  |  |
| Ophioglossum petiolatum |  | 1 |  |  |  |  |  |  |  |  |  |  |  |  |  |  |  |  |  |  |  |  |  |  |  |  |  |  |  |  |  |  |
| Adenostemma lavenia |  |  |  |  |  | 1 |  |  |  |  |  |  |  |  |  |  |  |  |  |  |  |  |  |  |  |  |  |  |  |  |  |  |
| Rheum palmatum |  |  |  |  |  |  |  |  |  |  |  |  |  |  |  |  |  |  |  |  |  |  |  |  |  |  |  |  |  |  | 1 |  |
| Crataegus pinnatifida |  |  |  |  |  |  |  |  |  |  | 1 |  |  |  |  |  |  |  |  |  |  |  |  |  |  |  |  |  |  |  |  |  |
| Salvia miltiorrhiza |  |  |  |  |  |  |  |  |  |  | 1 |  |  |  |  |  |  |  |  |  |  |  |  |  |  |  |  |  |  |  |  |  |
| Schisandra chinensis |  |  |  |  |  |  |  |  |  |  |  |  |  |  |  |  |  |  |  |  |  |  |  |  |  |  |  |  |  |  | 1 |  |
| Ludwigia octovalvis |  |  |  |  |  |  |  |  |  |  |  |  |  |  |  |  |  |  |  | 1 |  |  |  |  |  |  |  |  |  |  |  |  |
| Imperata cylindrica |  |  |  |  |  |  |  |  |  |  |  |  |  |  |  | 1 |  |  |  |  |  |  |  |  |  |  |  |  |  |  |  |  |
| Senna tora |  | 1 |  |  |  |  |  |  |  |  |  |  |  |  |  |  |  |  |  |  |  |  |  |  |  |  |  |  |  |  |  |  |
| Plantago asiatica |  |  |  |  |  |  |  |  |  |  |  |  |  |  |  |  |  | 1 |  |  |  |  |  |  |  |  |  |  |  |  |  |  |
| Eleutherococcus senticosus |  |  |  |  |  |  |  |  |  |  | 1 |  |  |  |  |  |  |  |  |  |  |  |  |  |  |  |  |  |  |  |  |  |
| Angelica keiskei |  |  |  |  |  |  |  |  |  |  |  |  |  |  |  |  |  |  |  |  | 1 |  |  |  |  |  |  |  |  |  |  |  |
| Lonicera japonica |  |  |  |  |  |  |  |  |  |  |  |  |  |  |  |  |  |  |  |  |  | 1 |  |  |  |  |  |  |  |  |  |  |
| Alternanthera sessilis |  |  |  |  |  |  |  |  |  |  |  |  |  |  |  |  |  |  |  |  |  |  |  |  |  | 1 |  |  |  |  |  |  |
| Kadsura japonica |  |  | 1 |  |  |  |  |  |  |  |  |  |  |  |  |  |  |  |  |  |  |  |  |  |  |  |  |  |  |  |  |  |
| Glossocardia bidens |  |  |  |  |  |  |  |  |  |  |  |  |  |  |  | 1 |  |  |  |  |  |  |  |  |  |  |  |  |  |  |  |  |
| Uncaria hirsuta |  |  |  |  |  | 1 |  |  |  |  |  |  |  |  |  |  |  |  |  |  |  |  |  |  |  |  |  |  |  |  |  |  |
| Prunella vulgaris |  |  |  |  | 1 |  |  |  |  |  |  |  |  |  |  |  |  |  |  |  |  |  |  |  |  |  |  |  |  |  |  |  |
| Bupleurum chinense |  |  |  |  |  |  |  |  |  |  | 1 |  |  |  |  |  |  |  |  |  |  |  |  |  |  |  |  |  |  |  |  |  |
| Gardenia jasminoides |  |  |  |  |  |  |  |  | 1 |  |  |  |  |  |  |  |  |  |  |  |  |  |  |  |  |  |  |  |  |  |  |  |
| Stevia rebaudiana |  |  |  |  |  |  |  |  |  |  |  |  |  |  |  | 1 |  |  |  |  |  |  |  |  |  |  |  |  |  |  |  |  |
| Citrus reticulata |  |  |  |  |  |  |  |  |  |  | 1 |  |  |  |  |  |  |  |  |  |  |  |  |  |  |  |  |  |  |  |  |  |
| Pogonatherum crinitum |  |  |  |  |  |  |  |  |  |  |  |  |  |  |  |  |  |  |  |  |  |  |  |  |  |  |  |  |  | 1 |  |  |
| Perilla frutescens |  |  |  | 1 |  |  |  |  |  |  |  |  |  |  |  |  |  |  |  |  |  |  |  |  |  |  |  |  |  |  |  |  |
| Astragalus propinquus |  |  |  |  |  |  |  |  |  |  | 1 |  |  |  |  |  |  |  |  |  |  |  |  |  |  |  |  |  |  |  |  |  |
| Coptis chinensis |  |  |  |  |  |  |  |  |  |  |  |  |  |  |  |  |  |  |  |  |  |  |  |  |  |  |  |  |  |  | 1 |  |
| Phyllanthus urinaria |  |  |  |  |  |  |  |  |  |  |  |  |  |  |  |  |  |  |  |  | 1 |  |  |  |  |  |  |  |  |  |  |  |
| Centella asiatica |  |  |  |  |  |  |  |  |  |  |  |  |  |  |  |  |  |  |  |  |  |  |  |  | 1 |  |  |  |  |  |  |  |
| Onychium japonicum |  |  |  |  | 1 |  |  |  |  |  |  |  |  |  |  |  |  |  |  |  |  |  |  |  |  |  |  |  |  |  |  |  |
| Juncus effusus |  |  |  |  |  |  |  |  |  |  |  |  |  |  |  |  |  |  |  | 1 |  |  |  |  |  |  |  |  |  |  |  |  |
| Solanum americanum |  |  |  |  |  |  |  |  |  |  |  |  |  |  |  |  |  | 1 |  |  |  |  |  |  |  |  |  |  |  |  |  |  |
| Curcuma longa |  |  |  |  |  |  |  |  |  |  |  |  |  |  |  |  |  |  |  |  | 1 |  |  |  |  |  |  |  |  |  |  |  |
| Cirsium brevicaule |  |  |  |  |  |  |  |  |  |  |  |  |  |  |  |  |  |  |  |  |  | 1 |  |  |  |  |  |  |  |  |  |  |
| Codonopsis pilosula |  |  |  |  |  |  |  |  |  |  | 1 |  |  |  |  |  |  |  |  |  |  |  |  |  |  |  |  |  |  |  |  |  |
| Ganoderma lucidum |  |  |  |  |  |  |  |  |  |  |  |  |  |  |  |  |  |  |  |  | 1 |  |  |  |  |  |  |  |  |  |  |  |
| Curcuma aromatica |  |  |  |  |  |  |  |  |  |  | 1 |  |  |  |  |  |  |  |  |  |  |  |  |  |  |  |  |  |  |  |  |  |
| ^a^N, northern Taiwan; ^b^C, central Taiwan; ^c^S, southern Taiwan; ^d^E, eastern Taiwan. | | | | | | | | | | | | | | | | | | | | | | | | | | | | | | | | |

| **Table S3 Medicinal materials with their UV and *Pi* for Taiwanese bitter tea in the three areas of Taiwan.** | | | | | | | | | | | |
| --- | --- | --- | --- | --- | --- | --- | --- | --- | --- | --- | --- |
| **Nothern** | **UV^a^** | ***Pi^b^*** | ***log_2_Pi*** | **Central** | **UV** | ***Pi*** | ***log_2_Pi*** | **Southern** | **UV** | ***Pi*** | ***log_2_Pi*** |
| Tithonia diversifolia (stem) | 0.47 | 0.073 | -3.78 | Ajuga nipponensis | 0.44 | 0.082 | -3.61 | Platostoma palustre | 0.50 | 0.143 | -2.81 |
| Andrographis paniculata | 0.47 | 0.073 | -3.78 | Bombax ceiba | 0.44 | 0.082 | -3.61 | Tithonia diversifolia (stem) | 0.33 | 0.095 | -3.39 |
| Ilex asprella | 0.40 | 0.063 | -4.00 | Andrographis paniculata | 0.22 | 0.041 | -4.61 | Ixeris chinensis | 0.33 | 0.095 | -3.39 |
| Ajuga nipponensis | 0.33 | 0.052 | -4.26 | Ixeris chinensis | 0.22 | 0.041 | -4.61 | Rhinacanthus nasutus | 0.33 | 0.095 | -3.39 |
| Ixeris chinensis | 0.27 | 0.042 | -4.58 | Ilex asprella | 0.22 | 0.041 | -4.61 | Orthosiphon aristatus | 0.33 | 0.095 | -3.39 |
| Solanum incanum | 0.27 | 0.042 | -4.58 | Mallotus repandus | 0.22 | 0.041 | -4.61 | Glycyrrhiza uralensis | 0.33 | 0.095 | -3.39 |
| Sigesbeckia orientalis | 0.27 | 0.042 | -4.58 | Physalis angulate | 0.22 | 0.041 | -4.61 | Andrographis paniculata | 0.17 | 0.048 | -4.39 |
| Bombax ceiba | 0.20 | 0.031 | -5.00 | Boehmeria nivea | 0.22 | 0.041 | -4.61 | Solanum incanum | 0.17 | 0.048 | -4.39 |
| Tithonia diversifolia (leaf) | 0.20 | 0.031 | -5.00 | Scutellaria barbata | 0.22 | 0.041 | -4.61 | Mallotus repandus | 0.17 | 0.048 | -4.39 |
| Rhinacanthus nasutus | 0.13 | 0.021 | -5.58 | Oldenlandia diffusa | 0.22 | 0.041 | -4.61 | Momordica charantia | 0.17 | 0.048 | -4.39 |
| Mallotus repandus | 0.13 | 0.021 | -5.58 | Bidens pilosa | 0.22 | 0.041 | -4.61 | Bidens pilosa | 0.17 | 0.048 | -4.39 |
| Glycyrrhiza uralensis | 0.13 | 0.021 | -5.58 | Tithonia diversifolia (stem) | 0.11 | 0.020 | -5.61 | Sphagneticola calendulacea | 0.17 | 0.048 | -4.39 |
| Boehmeria nivea | 0.13 | 0.021 | -5.58 | Solanum incanum | 0.11 | 0.020 | -5.61 | Pteris multifida | 0.17 | 0.048 | -4.39 |
| Momordica charantia | 0.13 | 0.021 | -5.58 | Rhinacanthus nasutus | 0.11 | 0.020 | -5.61 | Sophora flavescens | 0.17 | 0.048 | -4.39 |
| Mucuna macrocarpa | 0.13 | 0.021 | -5.58 | Orthosiphon aristatus | 0.11 | 0.020 | -5.61 | Morus alba | 0.17 | 0.048 | -4.39 |
| Gynostemma pentaphyllum | 0.13 | 0.021 | -5.58 | Platostoma palustre | 0.11 | 0.020 | -5.61 | Phellodendron chinense | 0.17 | 0.048 | -4.39 |
| Ocimum gratissimum | 0.13 | 0.021 | -5.58 | Mucuna macrocarpa | 0.11 | 0.020 | -5.61 | Mentha arvensis | 0.17 | 0.048 | -4.39 |
| Odontosoria chinensis | 0.13 | 0.021 | -5.58 | Sphagneticola calendulacea | 0.11 | 0.020 | -5.61 | Alternanthera sessilis | 0.17 | 0.048 | -4.39 |
| Solanum violaceum | 0.13 | 0.021 | -5.58 | Pteris multifida | 0.11 | 0.020 | -5.61 | Pogonatherum crinitum | 0.17 | 0.048 | -4.39 |
| Artemisia capillaris | 0.13 | 0.021 | -5.58 | Elephantopus scaber | 0.11 | 0.020 | -5.61 | Centella asiatica | 0.17 | 0.048 | -4.39 |
| Taraxacum campylodes | 0.13 | 0.021 | -5.58 | Salvia plebeian | 0.11 | 0.020 | -5.61 |  |  |  |  |
| Terminalia catappa | 0.13 | 0.021 | -5.58 | Ludwigia octovalvis | 0.11 | 0.020 | -5.61 |  |  |  |  |
| Physalis angulate | 0.07 | 0.010 | -6.58 | Imperata cylindrical | 0.11 | 0.020 | -5.61 |  |  |  |  |
| Orthosiphon aristatus | 0.07 | 0.010 | -6.58 | Plantago asiatica | 0.11 | 0.020 | -5.61 |  |  |  |  |
| Scutellaria barbata | 0.07 | 0.010 | -6.58 | Angelica keiskei | 0.11 | 0.020 | -5.61 |  |  |  |  |
| Oldenlandia diffusa | 0.07 | 0.010 | -6.58 | Lonicera japonica | 0.11 | 0.020 | -5.61 |  |  |  |  |
| Sphagneticola calendulacea | 0.07 | 0.010 | -6.58 | Glossocardia bidens | 0.11 | 0.020 | -5.61 |  |  |  |  |
| Pteris multifida | 0.07 | 0.010 | -6.58 | Stevia rebaudiana | 0.11 | 0.020 | -5.61 |  |  |  |  |
| Elephantopus scaber | 0.07 | 0.010 | -6.58 | Phyllanthus urinaria | 0.11 | 0.020 | -5.61 |  |  |  |  |
| Salvia plebeia | 0.07 | 0.010 | -6.58 | Juncus effuses | 0.11 | 0.020 | -5.61 |  |  |  |  |
| Morus alba | 0.07 | 0.010 | -6.58 | Solanum americanum | 0.11 | 0.020 | -5.61 |  |  |  |  |
| Mentha arvensis | 0.07 | 0.010 | -6.58 | Curcuma longa | 0.11 | 0.020 | -5.61 |  |  |  |  |
| Ophioglossum petiolatum | 0.07 | 0.010 | -6.58 | Cirsium brevicaule | 0.11 | 0.020 | -5.61 |  |  |  |  |
| Adenostemma lavenia | 0.07 | 0.010 | -6.58 | Ganoderma lucidum | 0.11 | 0.020 | -5.61 |  |  |  |  |
| Crataegus pinnatifida | 0.07 | 0.010 | -6.58 |  |  |  |  |  |  |  |  |
| Salvia miltiorrhiza | 0.07 | 0.010 | -6.58 |  |  |  |  |  |  |  |  |
| Senna tora | 0.07 | 0.010 | -6.58 |  |  |  |  |  |  |  |  |
| Eleutherococcus senticosus | 0.07 | 0.010 | -6.58 |  |  |  |  |  |  |  |  |
| Kadsura japonica | 0.07 | 0.010 | -6.58 |  |  |  |  |  |  |  |  |
| Uncaria hirsuta | 0.07 | 0.010 | -6.58 |  |  |  |  |  |  |  |  |
| Prunella vulgaris | 0.07 | 0.010 | -6.58 |  |  |  |  |  |  |  |  |
| Bupleurum chinense | 0.07 | 0.010 | -6.58 |  |  |  |  |  |  |  |  |
| Gardenia jasminoides | 0.07 | 0.010 | -6.58 |  |  |  |  |  |  |  |  |
| Citrus reticulata | 0.07 | 0.010 | -6.58 |  |  |  |  |  |  |  |  |
| Perilla frutescens | 0.07 | 0.010 | -6.58 |  |  |  |  |  |  |  |  |
| Astragalus propinquus | 0.07 | 0.010 | -6.58 |  |  |  |  |  |  |  |  |
| Onychium japonicum | 0.07 | 0.010 | -6.58 |  |  |  |  |  |  |  |  |
| Codonopsis pilosula | 0.07 | 0.010 | -6.58 |  |  |  |  |  |  |  |  |
| Curcuma aromatica | 0.07 | 0.010 | -6.58 |  |  |  |  |  |  |  |  |
| ^a^ UV, use value; ^b^ *Pi*, the proportion of the individual number of the *i*th medicinal material to the total number of medicinal material individuals. | | | | | | | | | | | |
